# Reliability and Agreement of a Dual-Method Radiographic Standard vs. Clinical Goniometry for Shank–Forefoot Alignment: A GRRAS-Compliant Study

**DOI:** 10.3390/diagnostics16050703

**Published:** 2026-02-27

**Authors:** Sergi Carrelero-Camp, Miki Dalmau-Pastor, Vanessa Oliva-Garballo, Clara Simón-de Blas, Carles Vergés-Sala, Elena de Planell-Mas

**Affiliations:** 1 Departmental Section of Podiatry, Department of Clinical Sciences, School of Medicine and Health Sciences, University of Barcelona, 08907 Barcelona, Spain; vanessaoliva_79@ub.edu (V.O.-G.); cverges@ub.edu (C.V.-S.); elenaplanell@ub.edu (E.d.P.-M.); 2 Human Anatomy and Embryology Unit, Department of Pathology and Experimental Therapeutics, School of Medicine and Health Sciences, University of Barcelona, 08907 Barcelona, Spain; mikeldalmau@ub.edu; 3 MIFAS by GRECMIP (Minimally Invasive Foot and Ankle Society), F-33700 Merignac, France; 4 Computer Science and Statistics Department, ETSII, Rey Juan Carlos University, 28933 Madrid, Spain; clara.simon@urjc.es

**Keywords:** forefoot varus, shank-forefoot alignment, reproducibility of results, radiography, goniometry, reference standards

## Abstract

**Introduction**: Forefoot varus prevalence varies widely (8.6–83.67%) and may be attributable to the lack of a gold standard. Clinical methods are limited by subjective positioning and landmark variability. This study established a reliable radiographic reference using a dual-method approach. **Methods**: Following GRRAS guidelines, 70 lower limbs (35 participants) were evaluated using consecutive sampling. Three blinded investigators performed the measurements. Reliability was assessed throughout inter-rater (30-min interval) and intra-rater (≥30-day washout) sessions. Dual radiographic methods (metallic nail vs. radiopaque markers) were compared against standardized goniometry. The analysis included ICC(2,1) and ICC(3,1), SEM, MDC_95_, and Bland–Altman plots, with a significance threshold of *p* < 0.005. A sensitivity analysis using one randomly selected limb per participant (n = 35) confirmed the robustness of the findings. **Results**: Participants (mean age 32.77 ± 10.8 years, 54% female) had a mean forefoot varus of 15.53 ± 7.35°, with a prevalence of 97.1% (≥0°). Goniometric inter-rater reliability was excellent (ICC = 0.987, MDC_95_ = 1.382°). The marker-based radiographic method demonstrated excellent intra-rater reliability (ICC = 0.906, MDC_95_ = 1.129°). The agreement between goniometry and marker-based radiography was strong (ICC = 0.898). All analyses surpassed the *p* < 0.005 threshold. No significant differences were found for sex or laterality (*p* > 0.005). **Conclusions**: Marker-based radiography provides a validated reference standard with excellent reliability. Standardized clinical goniometry demonstrated excellent reliability and strong radiographic agreement, making it appropriate for routine assessment (changes > 1.4° represent true structural change). Radiography should be reserved for research or definitive structural confirmation. Limitations include the lack of inter-reader radiographic reproducibility assessment and limited generalizability to young adults (mean age 33 years) with predominantly forefoot varus alignment.

## 1. Introduction

Forefoot varus (FV) is a structural/functional foot deformity characterized by inversion of the forefoot relative to the rearfoot [1]. FV is clinically considered a malalignment when the forefoot is inverted relative to the rearfoot by ≥8° in the non-weight-bearing subtalar neutral position [2,3]. Some studies have demonstrated significant relationships between forefoot varus and increased pronation-related parameters. Silva et al. found that forefoot varus predicts subtalar hyperpronation in young individuals [4], whereas Monaghan et al. reported that the forefoot angle determines the duration and amplitude of pronation during walking [5]. Additionally, forefoot varus has been associated with patellar tendinopathy in athletes [6]. In a cadaveric study, Lufler et al. demonstrated a significant association between forefoot varus deformity and patellofemoral cartilage damage, specifically affecting the inferomedial patellar facet [7]. The authors proposed that forefoot varus induces excessive foot pronation, generating femoral and tibial internal rotation that medially displaces the inferior pole of the patella, causing the inferomedial patellar facet to impact against the medial femoral trochlea, resulting in compartment-specific cartilaginous damage (91% prevalence in varus specimens versus 54% in valgus) [7].

However, other investigations have yielded inconsistent findings during gait analysis. Paes et al. demonstrated that clinical shank–forefoot alignment measurements only partially reflect the mechanical properties of the midfoot joint complex, suggesting that traditional static forefoot assessments may not fully capture dynamic biomechanical behavior [8]. During walking, Magalhães et al. found that midfoot passive stiffness, rather than forefoot alignment per se, was the primary factor affecting foot and ankle kinematics and kinetics during the propulsive phase [9]. Similarly, Cardoso et al. reported that hip external rotation stiffness and midfoot mechanical resistance were the dominant factors associated with lower limb movement during gait, with forefoot alignment showing limited predictive value for dynamic function [10]. This inconsistency in the literature may partially reflect limitations in measurement reliability and validity, underscoring the importance of establishing standardized, validated assessment protocols [11].

The reported prevalence of FV varies substantially across studies, ranging from 8.6% to 83.67% [12,13]. This wide range is attributable to population variation and the lack of standardized measurement protocols and diagnostic thresholds.

Two fundamental challenges persist in FV assessment. First, there is no universally accepted ‘gold standard’ measurement method, creating significant clinical uncertainty and hindering the comparison of research findings [11]. Second, diagnostic thresholds vary substantially across the literature, with some studies reporting clinically significant FV at different cut-off values and measurement methods [14,15,16]. This lack of consensus regarding both measurement techniques and diagnostic criteria has contributed to the wide prevalence ranges observed in the literature.

Current clinical assessment methods, including goniometric and photogrammetric techniques, demonstrate variable reliability depending on the specific protocol employed. Carrelero Camp et al. [11] found that although photogrammetric and shank–forefoot alignment methods demonstrate excellent reliability (ICC > 0.90), traditional goniometric forefoot alignment shows inconsistent, moderate inter-rater reliability (ICCs 0.56–0.68) when performed without standardized protocols. These techniques typically measure shank–forefoot alignment or isolated forefoot alignment, with methods depending on surface landmarks that may be susceptible to measurement variability [16,17]. Radiographic methods offer the potential for superior anatomical precision by directly visualizing skeletal structures, but their clinical utility and methodological performance require comprehensive validation.

This study proposes and validates a dual-method radiographic approach for FV assessment. We adapted two complementary techniques: the original shank–forefoot nail method for radiographic measurement [16], using a metallic nail under the metatarsal heads (nail), and a modified protocol using external radiopaque markers at the first and fifth metatarsal heads (mark). Both methods provide direct visualization of skeletal landmarks while maintaining clinical feasibility. By validating these radiographic approaches against standardized clinical goniometry, we aimed to establish anatomically precise yet clinically practical measurement protocols applicable across the spectrum of deformity severity.

### 1.1. Primary Objective

The primary aim of this study was to quantify the intra- and inter-rater reliability and inter-method agreement of standardized clinical and radiographic techniques for assessing forefoot varus alignment, addressing persistent methodological limitations and variability in forefoot measurement practices.

### 1.2. Exploratory Objective

As a secondary exploratory analysis, we examined the association between shank–forefoot alignment (SFA) and the static foot posture index (FPI). The FPI may reflect normal anatomical variation, adaptive postural responses, or both, and should be interpreted within the broader clinical context rather than as definitive evidence of pathological compensation.

## 2. Materials and Methods

### 2.1. Participants

A consecutive sampling approach was employed to enhance ecological validity and minimize selection bias. Volunteers were recruited from “Hospital Podològic Universitat de Barcelona” (managed by Fundació Josep Finestres) between January 2024 and May 2024. Inclusion criteria were age > 18 years old, ability to actively maintain ankle dorsiflexion at 90°, and willingness to participate in both goniometric and radiographic measurement protocols. A total of 35 participants were enrolled (54% females; age 32.7 ± 10.8; BMI 23.3 ± 3.3 kg·m^−2^), yielding a total of 70 lower limbs for analysis. For the intra-rater reliability assessment, the subsample of 23 participants was determined based on similar calculations to achieve adequate precision for ICC estimation with 95% confidence intervals [18]. Based on standardized goniometric assessment, 68 limbs (97.1%) were classified as forefoot varus, whereas 2 limbs (2.9%) exhibited forefoot valgus. Regarding habitual footwear and orthotic use, 20% of the participants (n = 14) reported regular use of barefoot shoes and 14.3% (n = 10) reported plantar orthoses.

### 2.2. Study Design

A cross-sectional methodological reliability study assessing the reliability and agreement of a radiographic measurement technique against a standard goniometric assessment was performed. This reliability study was conducted in accordance with the Guidelines for Reporting Reliability and Agreement Studies (GRRAS) [19].

Participants completed a single session that included both goniometric and radiographic assessment. Goniometric measurements were performed independently by two evaluators in a randomized order, with a 30-min interval between assessments. The evaluators were classified according to their experience with the standardized goniometric protocol: expert (S.C 15 years with similar protocols) and novice (E.M no prior experience with this specific protocol despite 20+ years of clinical podiatric experience). All evaluators completed a standardized 5-hour training session before data collection. Subsequently, all radiographic measurements were obtained by a certified radiographic specialist (V.O).

The evaluators were blinded to each other’s measurements, and identical goniometers that were calibrated prior to the study were used throughout. Statistical analyses were conducted by an independent analyst (C.S) blinded to evaluator identity and measurement outcomes.

### 2.3. Data Acquisition

#### 2.3.1. Intra-Rater Reliability

Intra-rater reliability was assessed on a sub-sample of 23 patients to evaluate consistency for both goniometric and radiographic measurements. The expert assessor performed a second set of goniometric measurements after a minimum one-month washout period to minimize recall bias. The radiologist repeated radiographic assessments on the same sub-sample after a minimum one-month interval, consistent with the washout period used for goniometric measurements.

#### 2.3.2. Goniometric Assessment

**Figure 1:**
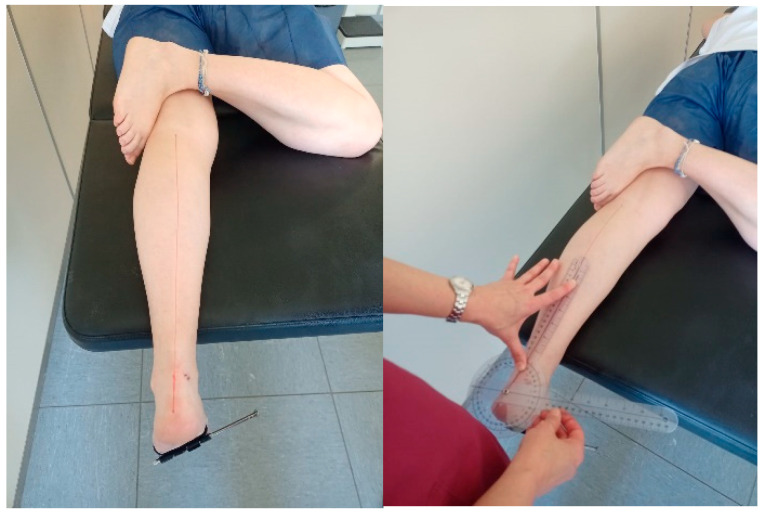
Goniometric assessment of shank–forefoot alignment.

Shank–forefoot alignment was assessed following Mendonça et al. [16] using a standard two-arm 50 cm goniometer (Miskall). Each evaluator performed three consecutive measurements per subject, repositioning the goniometer between measurements. The mean of the three measurements was calculated. This approach follows standard recommendations to reduce random measurement error [20]. The participants were asked to lie in prone position with the ankle at 90° dorsiflexion, placing a metallic nail under the metatarsal heads as a reference for the goniometer’s mobile arm, and aligning the stationary arm with the tibial bisector (Figure 1).

Static foot posture was assessed using the FPI, which is a validated clinical tool. The FPI quantifies posture through the evaluation of six distinct criteria: talar head palpation, supra and infra-lateral malleolar curvature, calcaneal frontal plane position, talonavicular joint prominence, medial longitudinal arch congruence, and forefoot-to-rearfoot alignment. Each criterion was scored on a scale from −2 to +2. The individual scores were summed to yield a total FPI score for each foot, which was then used to classify posture as supinated, neutral, or pronated, according to established clinical ranges [21].

#### 2.3.3. Radiographic Assessment

**Figure 2:**
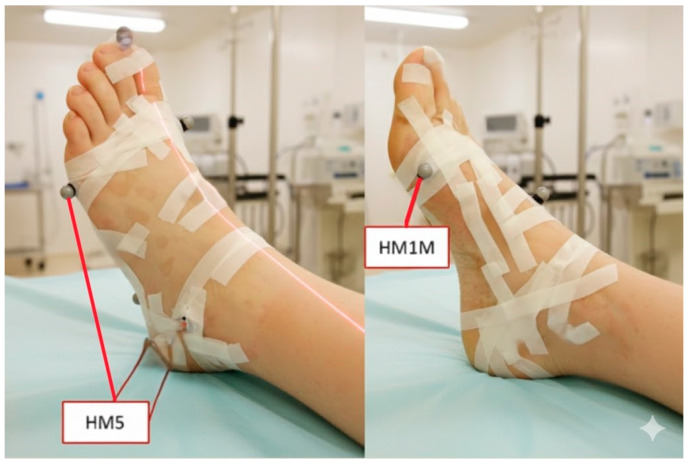
The placement of markers (HM1M and HM5) for radiographic measurement [25].

Radiographic assessments were conducted using the Siemens POLYMOBIL III/Plus system with VistaScan Plus Ceph software (Durr Dental) for measuring the shank–forefoot angle. Radiographs were obtained using standardized settings: 60 kV, 2.5 mA, and 0.1 exposure time. The source-to-image distance was fixed at 100 cm for all acquisitions. This study employed two distinct radiographic measurement techniques for comparative validation. First, a marker-based method (Marker method) is proposed as the reference standard. Two radiopaque markers were placed at the midpoint of the first metatarsal head (medial) and fifth metatarsal head (lateral), as described by the Heidelberg foot measurement protocol [22,23,24,25] (Figure 2).

**Figure 3:**
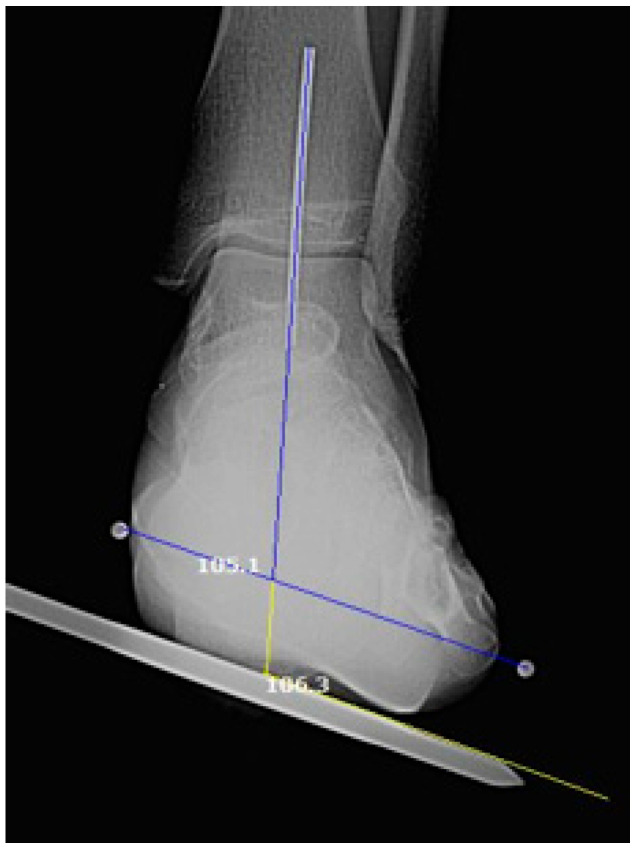
Radiographic assessment with the markers and nail.

Secondly, the nail-based method consists of placing a metallic nail under the metatarsal heads, which serves both as a reference landmark for goniometric measurement and as a radiopaque marker for radiographic assessment (Figure 3). Both methods measured the angle between the tibial bisector and the forefoot reference line. Each method was assessed at baseline (T1) (n = 70) and at 1-month (T2) (n = 23).

The tibial bisector was identified, and a reference line (with a second radiopaque pin) was placed along this axis. The angle between the tibial bisector and a line connecting the two markers (1st and 5th metatarsal heads) was defined as the shank–forefoot alignment angle. For comparative purposes, this angle was also independently calculated using the first radiopaque pin (used in the clinical measurement setup) as the forefoot reference point for comparative analysis. The radiographic approach was performed using markers placed on the medial side of the first metatarsal head and the lateral side of the fifth metatarsal head [25,26,27]. For observation of the tibial bisector (as performed in manual measurements), a reference line was placed along this axis, as shown in Figure 3. Due to ethical considerations regarding cumulative radiation exposure, each subject underwent a single radiographic acquisition per session.

### 2.4. Data Analysis

#### 2.4.1. Sample Size

The sample size calculation considered a minimally acceptable ICC (ρ_0_) of 0.65, an expected ICC (ρ_1_) of 0.85, a significance level (α) of 0.005, and a power (1 − β) of 80%, yielding a required sample size of 73 limbs. Our achieved sample of 70 limbs provided adequate statistical power for all planned analyses.

#### 2.4.2. Statistical Analysis

Data analysis was performed using IBM Corp. Released 2020. IBM SPSS Statistics for Windows, Version 27.0. Armonk, NY, USA: IBM Corp. and RStudio Team. RStudio: Integrated Development Environment for R. RStudio, PBC, Boston, MA, USA. Both limbs were analyzed to maximize statistical power, consistent with established practice in foot morphology reliability studies. No systematic laterality differences were observed between the sides (T = 0.22, *p* = 0.827). Although we acknowledge potential within-subject correlation between contralateral limbs, this methodological approach is widely accepted in reliability research focused on measurement precision rather than biological inference between individuals. For the agreement analysis, the modified radiographic method with radiopaque markers was considered the reference standard based on its theoretical superior precision and objective landmark identification, whereas goniometry served as the index test representing clinical practice. Reliability was assessed using intraclass correlation coefficients (ICCs) with 95% confidence intervals. For inter-rater goniometric reliability, intraclass correlation coefficients assuming evaluators were a random sample and estimating reliability for a single measurement or a single evaluator, ICC(2,1) was used. For intra-rater reliability, intraclass correlation coefficients with fixed evaluators and absolute consistency, ICC(3,1) was employed. Interpretation for the ICC values was considered as follows: values > 0.90 indicate excellent reliability; those between 0.75 and 0.90 indicate good reliability; those between 0.50 and 0.75 indicate moderate reliability, and values < 0.5 indicate low reliability [28]. Agreement between the methods was analyzed using Bland–Altman plots with 95% limits of agreement. The standard error of measurement (SEM) and minimal detectable change (MDC) were also calculated using the standard deviation of the differences between raters (SDdiff). SEM and MDC were computed using the following equations:

(1) $$SEM=SDdiff\sqrt{1-CCI}$$

and [29]

(2) $${MDC}_{95}=1.96\sqrt{2SEM}$$

The coefficient of variation (CV) was calculated as CV = (SD/Mean) × 100 to quantify relative measurement variability, where SD represents the standard deviation and mean represents the mean value of the measurements [29]. The CV was reported for individual measurements to facilitate comparison across different measurement scales. To assess the robustness of our findings to potential within-participant correlation arising from the inclusion of bilateral limb measurements, we conducted a sensitivity analysis for within-participant correlation using one randomly selected limb per participant (n = 35). For each participant, either the right or left limb was randomly selected using the random number generation function in Microsoft Excel (Microsoft Corporation, Redmond, WA, USA). Key reliability metrics (ICC, SEM, MDC_95_, and CV) were recalculated for this independent subsample to verify that the conclusions remained unchanged when accounting for the non-independence of bilateral observations. Pearson’s correlation coefficient (r) was used to study criterion validity between researchers, interpreting correlation coefficients from 0.00 to 0.49 as poor, those from 0.50 to 0.79 as moderate, and those of 0.80 or higher as excellent [30]. After assessing normality using the Kolmogorov–Smirnov test, mean group comparisons were conducted according to the experimental design. Student’s *t*-test was applied for normally distributed data, whereas the Mann–Whitney U test was used for non-parametric cases. A one factor ANOVA table was constructed to determine the existence of statistically significant differences among FPI scores and shank–forefoot measurements. Reliability coefficients are influenced by whether measurements represent single observations or averages of multiple observations [31]. In this study, goniometric ICC values reflect the reliability of mean scores (the average of three measurements per evaluator), whereas radiographic ICC values reflect single measurements. This methodological difference was necessary due to ethical constraints on radiation exposure. The comparison remains valid as both protocols represent their respective real-world clinical applications: goniometry allows multiple measurements without risk, whereas radiography is limited to single acquisitions in practice. The significance criterion was established to *p* < 0.005 instead of *p* < 0.05, as the latter has a considerably high probability (around 26%) of being a false positive. This change strengthens the robustness of the results [19]. We selected α = 0.005 to minimize false-positive findings, as recommended for improving reproducibility in scientific research [32]. However, all our key ICC values and statistical inferences remain robust and statistically significant at the conventional α = 0.05 level, with all primary ICC estimates exceeding established reliability thresholds (>0.90 for excellent reliability) regardless of the significance threshold applied. More information is provided in the Supplementary File S3.

## 3. Results

### 3.1. Sample Characteristics and Descriptive Statistics

**Figure 4:**
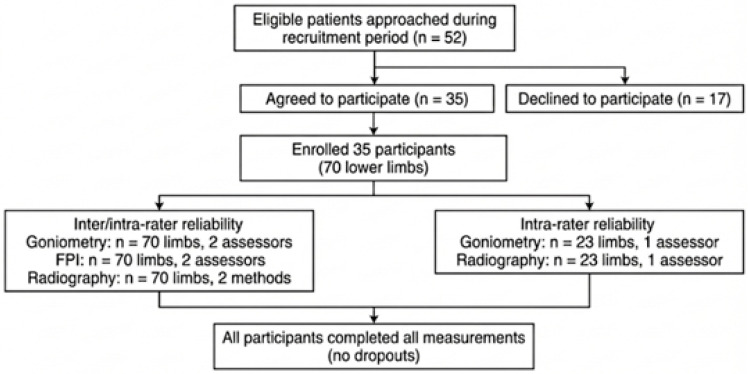
Participant flow diagram.

Seventy limbs from 35 participants were included in this study (Figure 4). Mean ± SD shank–forefoot alignment was 15.53 ± 7.35° (median: 16.0°, IQR: 10.0–22.0°, range: −1.0° to 29.7°). Forefoot varus (≥0°) was observed in 97.1% of limbs (68/70), with 52.9% (37/70) presenting values ≥ 15°.

### 3.2. Marks and Nails

Marks 1 (mean: 15.74 ± 7.88, range: −1.5 to 36.5) and 2 (mean: 15.08 ± 7.56, range: −8.4 to 30.1) and nails 1 (mean: 15.1 ± 8.7, range: −2.4 to 35.6), and 2 (mean: 14.97 ± 8.76, range: −10.3 to 30) measurements presented high positive and significant correlations (Table 1). The ICCs among observations indicate moderate reliability for individual measurements and good reliability for mean measurements (Table 2). MDC_95_ indicates that changes of less than six points could be random errors, whereas changes of six points or more likely reflect a true improvement or decline in balance for individual Mark 1 and 2 scores.

**Figure 5:**
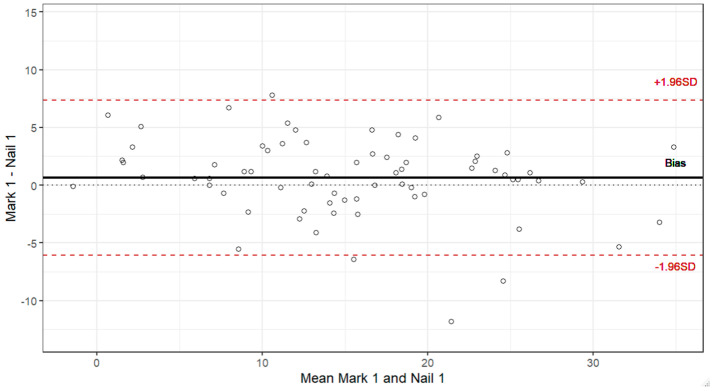

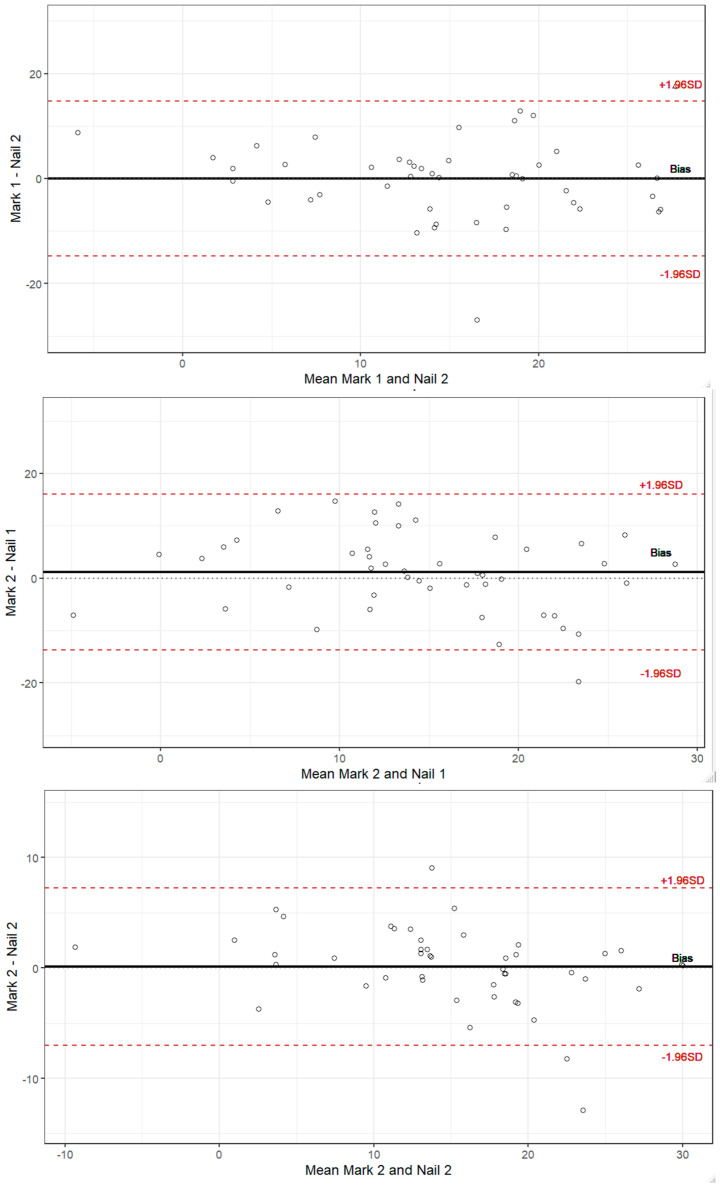
Bland–Altman plots for shank–forefoot measurements by Mark 1 and 2 and Nail 1 and 2.

The Kolmogorov–Smirnov test assumes normal distribution for the nail measurements (Znail 1 = 0.068; *p*-value = 0.577; Znail 2 = 0.096; *p*-value = 0.351) and Mark 1 (ZMark 1 = 0.091; *p*-value = 0.153), but failed for Mark 2 (ZMark 2 =0.154; *p*-value = 0.008). Bland–Altman plots for shank–forefoot measurements by Mark 1 and 2 and Nail 1 and 2 (Figure 5). No statistical differences were found by side or gender.

### 3.3. FPI

**Figure 6:**
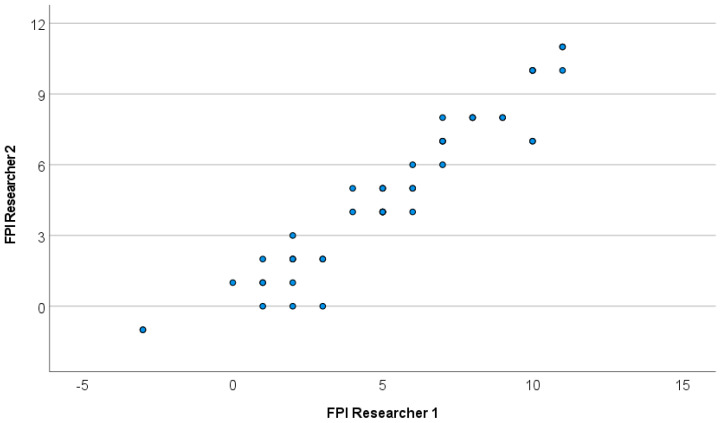
Scatter plot for FPI measurements by researcher.

No statistically significant differences were observed between the two measurements (χ^2^ = 253.19, *p*-value ≈ 0), and they showed a strong positive correlation (ρ = 0.953, *p*-value < 0.001) (Figure 6). The ICC among observations indicates excellent reliability for individual and mean measurements (Table 3). More information is provided in the Supplementary File S1.

### 3.4. Inter-Rater Reliability

The ICC among observations indicates excellent reliability for individual and mean measures (Table 4) and demonstrated a strong positive correlation (ρ = 0.987, *p*-value < 0.001). The Kolmogorov–Smirnov test assumes normal distribution for the shank–forefoot measurements (Z = 0.06; *p*-value = 0.762).

No differences were found by side (T-value = 0.22, *p*-value = 0.827 IC_95%_ (μ_Right_–μ_Left_) = [−3.22; 4.01]). Females (16.5 ± 7.35) exhibited higher values than males (13.39 ± 7.48), near the significance boundary (T-value = −1.75, *p*-value = 0.083 IC_95%_ (μ_Male_–μ_Female_) = [−6.68; 0.42]); therefore, a larger sample is recommended for further exploration of this finding. Barefoot shoes did not present significant differences in the shank–forefoot measurements (T-value = −1.51, *p*-value = 0.136 IC_95%_ (μ_No_–μ_Yes_) = [−7.81; 1.08]), nor did plantar orthotics (T-value = −0.405, *p*-value = 0.687 IC_95%_ (μ_No_–μ_Yes_) = [−4.11; 6.21]).

**Figure 7:**
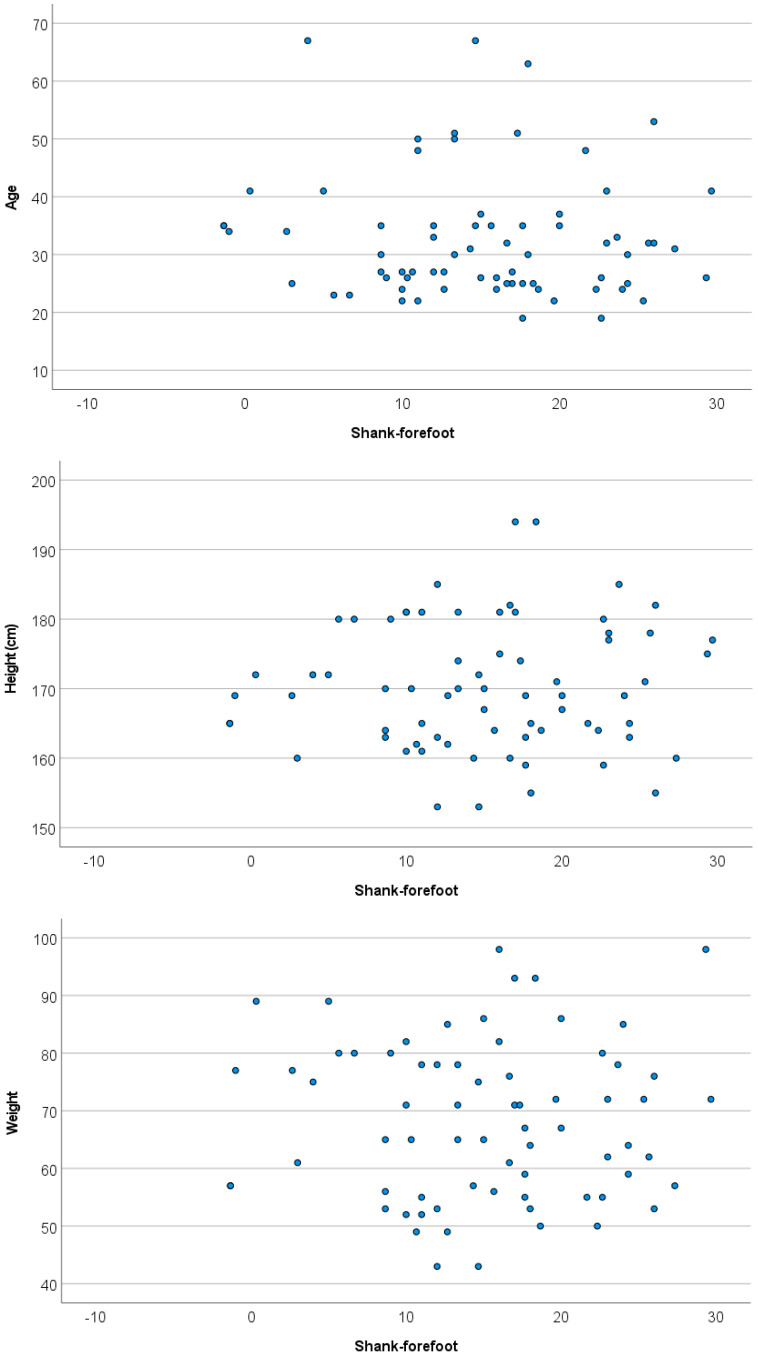

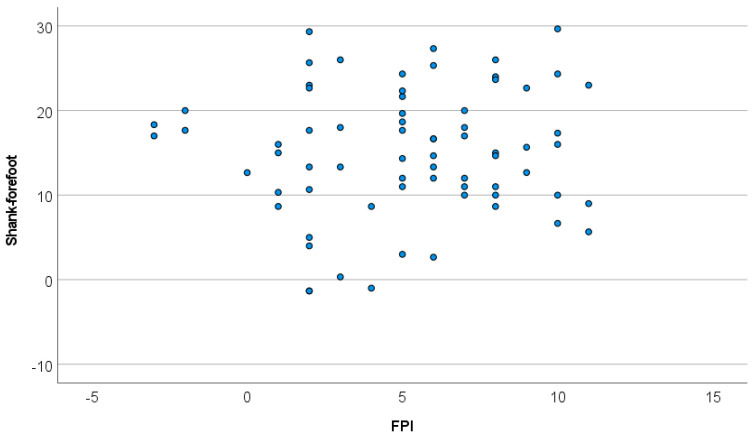
Scatter plots for age, height, weight, FPI and shank–forefoot measurements.

No relationship was observed among shank–forefoot measurement and the age, height, or weight of the patients (see Figure 7; ρ_weight_ = −0.001, *p*-value = 0.991; ρ_height_ = 0.06, *p*-value = 0.622; ρ_age_ = −0.082, *p*-value = 0.498) and FPI measurement (F-Snedecor = 0.579, *p*-value = 0.86).

The ICC among observations indicates moderate reliability for individuals and good reliability for mean measurements (Table 4). The bias was nearly 0%, almost constant across the entire range of the variable for Marks 1 and 2 and Nails 1 and 2. The limits of agreement ranged from 18% to −18% for Marks 1 and 2 and Nails 1 and 2 with respect to shank–forefoot measurements for Researcher 1.

**Figure 8:**
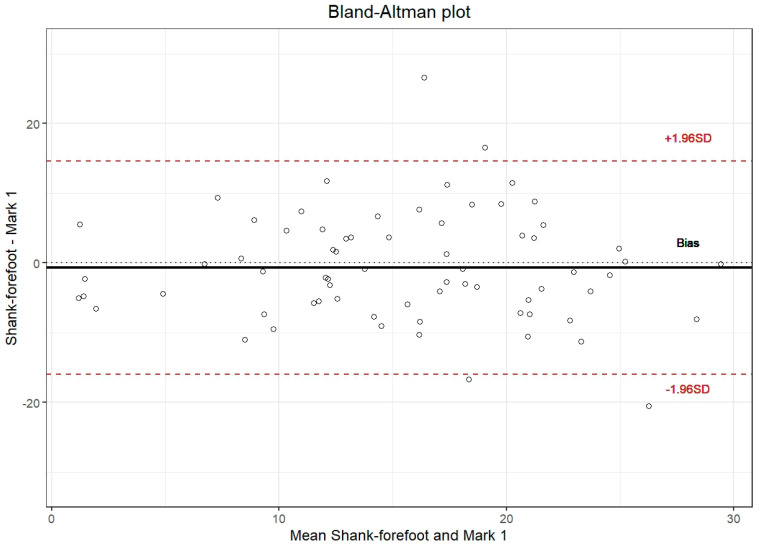

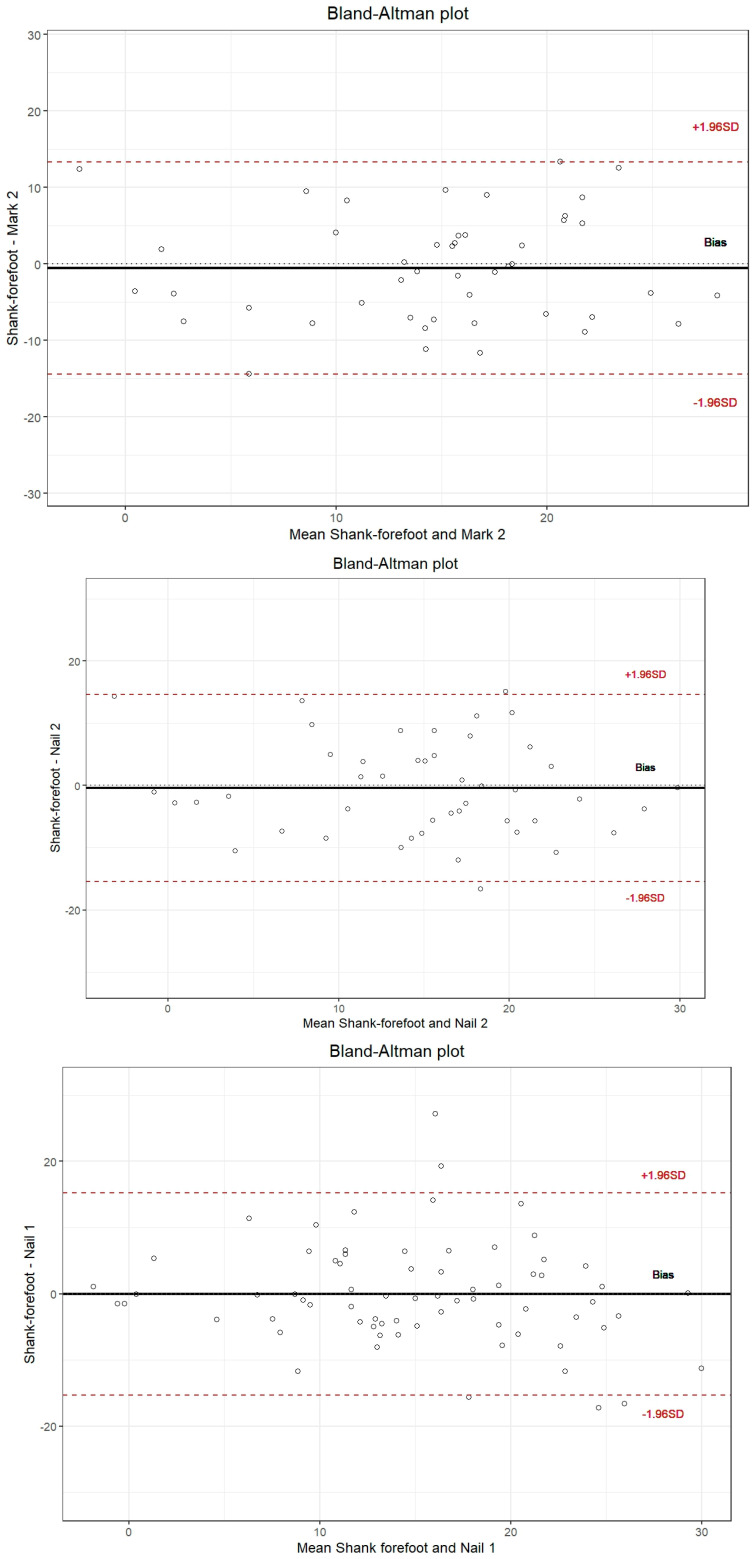
Bland–Altman plots for shank–forefoot measurements by Marks 1 and 2 and Nails 1 and 2.

The ICCs for Marks 1 and 2 and Nails 1 and 2 relatives to shank–forefoot measurements indicated moderate to low reliability for individual measurements and moderate reliability for mean measurements (Table 5). Bland–Altman plots for shank–forefoot measurements by Marks 1 and 2 and Nails 1 and 2 (Figure 8).

### 3.5. Inter-Rater/Intra-Rater Reliability (Secondary Measure)

The Kolmogorov–Smirnov test rejected normal distribution for the second shank–forefoot measurement (Z = 0.179; *p*-value = 0.001). The second shank–forefoot measurement exhibited a strong positive correlation with the first measurement (ρ_t_ = 0.869, *p*-value ≈ 0).

No differences were found by side (Mann–Whitney U = 231, *p*-value = 0.462) or gender (Mann–Whitney U = 319, *p*-value = 0.129). Barefoot shoes did not present significant differences in the shank–forefoot measurements (Mann–Whitney U = 290, *p*-value = 0.03), nor did plantar orthotics (U-Mann-Whitney = 10, *p*-value = 0.07). However, the values were near the significance boundary, so a larger sample may be recommended for further exploration of this finding.

No linear relationship was observed among the shank–forefoot second measurement and the age, height, and weight of the patients (ρ_weight_ = −0.048, *p*-value = 0.751; ρ_height_ = 0.128, *p*-value = 0.398; ρ_age_ = −0.148, *p*-value = 0.325). The FPI measurement (F-Snedecor = 2.81, *p*-value = 0.009) showed significant differences, with significantly smaller values for FPI 2 and 4 (5.09 ± 2.07 IC_95%_ (μ_2,4_) = [0.904, 9.28]) compared with the remaining values (15.93 ± 0.881 IC_95%_ (μ) = [14.16, 17.71]).

Detailed results are provided in the Supplementary File S2.

### 3.6. Sensitivity Analysis

The sensitivity analysis using one randomly selected limb per participant (n = 35) confirmed the robustness of all primary findings. Goniometric inter-rater reliability remained excellent (ICC(3,1) = 0.989, 95% CI [0.976; 0.995], MDC_95_ = 0.190°), as did marker-based radiographic intra-rater reliability (ICC(2,1) = 0.952, 95% CI [0.905; 0.976], MDC_95_ = 0.827°). Inter-method agreement between goniometry and marker-based radiography remained strong (ICC(3,1) = 0.933, 95% CI [0.878; 0.968], MDC_95_ = 1.130°). All ICC values remained in the excellent range, with negligible changes from the full dataset analysis (n = 70), and all statistical inferences remained significant at *p* < 0.005. Detailed results are provided in the Supplementary File S4.

## 4. Discussion

### 4.1. Principal Findings

The primary objective of this study was to establish a definitive radiographic reference standard to resolve the diagnostic uncertainty surrounding forefoot varus (FV), a condition that currently presents a wide and controversial prevalence range from 8.6% to 83.67% in the literature [12,13]. Our results successfully validated a dual-method radiographic approach, demonstrating that both metallic nails and radiopaque markers provide highly reliable measurements for establishing a gold standard. Furthermore, we confirmed that clinical goniometry, when performed under standardized shank–forefoot alignment (SFA) protocols, exhibits excellent agreement with this new radiographic reference, thereby providing clinicians with validated tools across different clinical contexts.

### 4.2. Reliability of the Radiographic Reference Standard

The marker-based radiographic method demonstrated excellent intra-rater reliability (ICC = 0.906, 95% CI: 0.852–0.944), exceeding the ICC > 0.90 threshold recommended for instruments used in individual clinical decision-making [19]. This level of precision is particularly critical given that this method serves as the reference standard against which clinical tools are validated. An MDC_95_ of 1.129° establishes a practical threshold below which observed changes likely represent measurement error rather than true structural change. Moreover, this precision is comparable to other established radiographic measurement protocols in foot and ankle biomechanics, such as the Heidelberg foot measurement method [25].

Although the metallic nail method showed a strong correlation with radiopaque markers (r = 0.919, *p* < 0.001), we observed greater variability in nail-based measurements (ICC = 0.784 for mean measurements compared to ICC = 0.906 for markers). This difference is likely attributable to positional instability when maintaining the ankle at 90° dorsiflexion, as the nail can shift with subtle changes in soft tissue tension or forefoot positioning. This finding aligns with previous observations that surface-based reference points are susceptible to soft tissue movement and positioning artifacts [17]. Consequently, we recommend the marker-based method as the superior radiographic standard when precision is paramount, though the nail method remains a viable alternative when marker placement is impractical or in settings where marginally lower precision is clinically acceptable.

### 4.3. Clinical Goniometry Performance and Protocol Improvements

The excellent inter-rater reliability of clinical goniometry using the standardized SFA protocol (ICC = 0.987, 95% CI: 0.977–0.993) represents a substantial improvement over traditional isolated forefoot goniometry, which typically yields ICCs of 0.56–0.68 [11]. This improvement can be attributed to three key modifications in the Mendonça protocol [16]: (1) elimination of subjective subtalar neutral positioning, which has demonstrated poor inter-rater reliability (ICC = 0.22–0.48) in previous studies [33,34]; (2) the use of a standardized metallic nail as a tactile reference for the mobile goniometer arm, reducing landmark identification variability, which can exceed 10° with traditional palpation methods [17]; and (3) strict 90° ankle positioning with clear anatomical criteria for identifying the tibial bisector. These protocol refinements effectively minimize the primary sources of measurement error that have historically plagued forefoot assessments.

Furthermore, the intra-rater reliability for goniometry (ICC = 0.857 for individual measurements, ICC = 0.923 for mean measurements) also demonstrated good to excellent consistency after a one-month washout period. The MDC_95_ of 1.382° for individual measurements indicates that changes of approximately 1.4° or greater likely represent true changes in forefoot alignment rather than measurement error. This threshold is particularly relevant for monitoring the progression of deformity or the response to conservative interventions, such as orthotic therapy or physical rehabilitation.

### 4.4. Inter-Rater Reliability and Normative Values

The excellent inter-rater reliability (ICC = 0.987) after only 5 h of novice training demonstrates exceptional protocol standardization. The mean difference between expert and novice raters was −0.21 ± 1.22° (*p* = 0.230), indicating negligible systematic bias, with 89.6% of measurements differing by <2°. These results substantially exceed traditional goniometric methods (ICC 0.56–0.68) [35] and confirm that brief standardized training is sufficient for clinical implementation. Our sample mean of 15.53 ± 7.35° (n = 70) is slightly higher than that of Mendonça et al. [16] (13.90 ± 9.88°, n = 400 limbs). This difference may be explained by our sample, in which forefoot varus was present in 97.1% of participants, compared to Mendonça’s more heterogeneous athletic population, where greater postural variability is expected.

### 4.5. Methodological Considerations in Comparing Goniometry and Radiography

Goniometric reliability (ICC = 0.987) was calculated from averaged measurements, whereas radiographic reliability (ICC = 0.906) relied on single acquisitions to minimize radiation exposure. Despite this, radiographic measurement achieves excellent reliability with a single acquisition, demonstrating superior measurement efficiency and validating its role as a structural reference standard. The strong agreement between averaged goniometric and single radiographic measurements (ICC = 0.898) confirms that standardized goniometry, when properly implemented with multiple measurements, provides valid clinical assessment, whereas radiography remains the gold standard for definitive structural quantification.

### 4.6. Agreement Between Clinical and Radiographic Methods

The strong agreement found between clinical goniometry and the marker-based radiographic standard (ICC = 0.898 for mean measurements) suggests that standardized clinical assessment is a valid proxy for osseous alignment in most clinical contexts. Bland–Altman analysis revealed that bias remained nearly constant (close to 0%) across the entire measurement range from −10° to 36°, confirming the absence of systematic errors or proportional bias. The 95% limits of agreement (±18% for most comparisons) indicate that although individual measurements may vary, the overall concordance is sufficient for clinical screening and decision-making.

However, it is important to note that previous research has documented a potential six-degree mean difference between clinical and digital photogrammetric methods [36], suggesting that practitioners should avoid using different methods interchangeably during longitudinal patient tracking. Although goniometry and radiography demonstrate strong agreement, MDC values indicate that inter-method switching may compromise the detection of meaningful clinical changes. Based on the present results, the consistent use of a single measurement method from baseline through follow-up is recommended to ensure reliable detection of progression and treatment effects.

For routine clinical screening, the excellent reliability and agreement of standardized goniometry (ICC = 0.987) validates its use as the primary assessment tool, avoiding unnecessary radiation exposure while maintaining measurement quality. Radiographic assessment should be reserved for research contexts requiring gold-standard precision, surgical planning where anatomical precision is critical, or cases where clinical findings are ambiguous and require definitive structural confirmation.

### 4.7. Demographic Variables and Population Characteristics

In accordance with our strict significance threshold of *p* < 0.005 [19], no statistically significant differences were found regarding sex or laterality. However, females presented clinically relevant higher SFA values (16.5° ± 7.35°) compared to males (13.39° ± 7.48°), with a difference of approximately 3° that approached but did not reach the significance threshold (*p* = 0.083). This three-degree difference exceeds the MDC_95_ of 1.382° for goniometric measurements, suggesting that it may represent a genuine structural difference rather than measurement error, despite lacking statistical significance in our sample. Similarly, the absence of significant differences between participants wearing barefoot shoes (20%, n = 14) versus conventional footwear (*p* = 0.136) or those using plantar orthotics (14.3%, n = 10) versus those without orthotics (*p* = 0.687) should be interpreted cautiously. These comparisons were underpowered and represent secondary exploratory analyses. Larger studies specifically designed to investigate the influence of footwear and orthotic interventions on forefoot structure would be needed to draw definitive conclusions.

### 4.8. Forefoot Varus and Foot Posture Index Relationship

Our study explored the biomechanical paradigm that non-weight-bearing osseous deformities drive weight-bearing postural compensations, as proposed by Root’s biomechanical theory [37]. We found a modest but significant correlation between FPI scores and the second radiographic nail measurement (r = 0.430, *p* = 0.003), supporting the hypothesis that forefoot varus may contribute to compensatory pronation patterns captured by the FPI. This relationship aligns with previous findings in young populations showing that forefoot varus predicts subtalar hyperpronation [4]. Critically, FPI was assessed only once (baseline), whereas SFA was measured twice. We cannot determine whether participants’ FPI classification remained stable across the 30-day interval. This temporal mismatch prevents definitive interpretation.

This finding highlights the critical distinction between static/functional osseous deformity (FV measured non-weight-bearing) and static postural compensation (FPI assessed weight-bearing). Although the biomechanical theory posits a causal relationship [37,38], the temporal variability observed in our study suggests that this relationship may be more complex and is potentially moderated by factors such as soft tissue flexibility, neuromuscular control, and foot type. Therefore, clinicians should assess both forefoot structure and functional foot posture as complementary but distinct clinical constructs.

### 4.9. Limitations

Despite these strengths, several limitations must be acknowledged. First, and most importantly, inter-rater reliability of the radiographic method was not assessed. Although we established excellent intra-rater reliability for the marker-based radiographic protocol, the reproducibility of measurements across different radiologists remains to be determined. This represents an important gap in establishing this method as a definitive reference standard. However, several methodological features support the expected generalizability of this digital, marker-based workflow across different readers:

- The measurement protocol relies on clearly visible radiopaque markers placed at anatomically distinct landmarks (1st and 5th metatarsal heads), minimizing subjective interpretation compared to methods requiring identification of subtle bony landmarks within radiographic images.
- The digital measurement workflow uses standardized software tools with objective angle calculations between marked reference points, eliminating manual protractor-based measurements that introduce operator-dependent variability.
- Previous validation studies of digital radiographic measurement systems have consistently demonstrated excellent inter-rater reliability (ICC > 0.90) when using marker-based or clearly defined anatomical reference points, suggesting that objective digital workflows reduce between-observer variation.

Nevertheless, formal evaluation of inter-reader reproducibility should be a high priority in future validation studies to definitively establish this method as a universally accepted reference standard. Until such validation is completed, clinicians and researchers should be aware that the generalizability of radiographic measurements across different operators has not been empirically demonstrated in this study.

Second, our sample was relatively homogeneous and young (mean age 32.77 ± 10.8 years, range 19–67), with the majority (97.1%) demonstrating forefoot varus rather than valgus alignment. This limits the generalizability of our findings to older adults, pediatric populations, or individuals with forefoot valgus deformities. The reliability and validity of these measurement protocols in older adults, individuals with forefoot valgus deformity, and patients with specific foot pathologies (e.g., hallux valgus, pes planus, or rheumatoid arthritis) remain to be established. Future studies should specifically recruit diverse populations, including older individuals, those with forefoot valgus, and patients with various foot and ankle pathologies, to determine whether these measurement protocols maintain their excellent reliability across different demographic and clinical groups. Age-related changes in foot structure, including decreased soft tissue flexibility and altered bone morphology, may affect both the magnitude of forefoot varus and the reliability of measurement methods. Additionally, our sample was recruited from a single academic podiatric hospital in Spain, potentially limiting geographic and cultural generalizability.

Third, both the radiographic and goniometric methods assessed forefoot alignment in non-weight-bearing positions with the ankle held at 90° dorsiflexion. Although this standardization improves reliability, it does not capture the dynamic behavior of the forefoot during weight-bearing activities or gait. Previous research has demonstrated that static assessments often fail to predict dynamic dysfunction [39], and the Framingham Foot Study specifically highlighted this discordance between static and dynamic measures. Therefore, the clinical relevance of non-weight-bearing forefoot varus measurements for predicting functional limitations or injury risk remains poorly understood. Future research incorporating dynamic assessment methods, such as 3D motion capture, would provide valuable complementary information. Although our radiographic approach provides high precision, the absolute anatomical gold standard remains direct visualization through cadaveric dissection [7,40]. Cadaveric models allow for the direct pinning of landmarks and cartilage scoring, effectively eliminating soft-tissue interference and imaging limitations [7]. Notably, Lufler et al. [40] found no significant association between clinical forefoot alignment and bony talar torsion (r = 0.18, *p* = 0.22), suggesting a soft-tissue origin rather than an unalterable bony deformity. Therefore, our in vivo protocol bridges the gap between these invasive anatomical findings and the need for a reproducible clinical standard.

Fourth, we analyzed both feet from each participant as independent observations after confirming no systematic laterality differences. Although we acknowledge the potential for within-participant correlation when including bilateral limbs, our sensitivity analysis using one randomly selected limb per participant (n = 35) confirmed that all primary findings and statistical inferences remained unchanged, supporting the validity of our conclusions.

Fifth, our study established the measurement reliability of these methods but did not assess their predictive validity for clinical outcomes. Establishing that a measurement is reliable is necessary but not sufficient for demonstrating clinical utility. Future longitudinal research should investigate whether specific forefoot varus thresholds predict the development of pathologies such as plantar fasciitis, posterior tibial tendon dysfunction, medial knee osteoarthritis, or other conditions hypothesized to result from compensatory pronation.

Finally, although we established the marker-based radiographic method as a reference standard based on theoretical superior precision and objective landmark identification, it remains a two-dimensional projection of three-dimensional structures. True gold-standard assessment would require direct anatomical measurement from cadaveric specimens (41). However, the practical and ethical constraints of these approaches make them infeasible for large-scale reliability studies in living subjects.

Despite these limitations, this study’s rigorous methodology (strict blinding, GRRAS compliance, and conservative significance threshold) and robust primary findings (excellent goniometric reliability and validated radiographic reference) provide a solid foundation for clinical application and future research.

### 4.10. Clinical Implications

The establishment of a validated radiographic reference standard, combined with demonstrated strong agreement with standardized clinical goniometry, provides important guidance for clinical practice. For routine clinical assessment and screening, practitioners can confidently employ the standardized SFA goniometric protocol [16] with the knowledge that measurements demonstrate excellent inter-rater reliability (ICC = 0.987) and strong agreement with radiographic gold standards. The MDC_95_ of 1.382° for goniometry provides a meaningful threshold for interpreting change: differences smaller than approximately 1.4° between repeated measurements likely represent measurement error rather than true structural change.

When research-grade precision is required, such as in clinical trials evaluating surgical outcomes, studies investigating the natural history of deformity progression, or cases where definitive structural assessment is needed for surgical planning, the marker-based radiographic method provides superior precision (MDC_95_ = 1.129°). However, the ionizing radiation exposure inherent to radiography limits its appropriateness for serial assessments in most clinical scenarios, particularly in pediatric populations or young adults who may require long-term monitoring.

Clinicians can use these reliable measurement methods to accurately quantify forefoot alignment. The excellent inter-rater reliability (ICC = 0.987) and low measurement error (MDC_95_ = 1.38°) ensure that serial measurements can detect true structural changes over time. Future research can use these validated protocols to investigate potential associations between forefoot alignment and lower extremity pathomechanics. Although forefoot and shank–forefoot alignment has been associated with various pathologies in previous literature, establishing these clinical associations was not within the scope of the current reliability study.

Clinicians should avoid switching between measurement methods (goniometry vs. radiography) when monitoring the same patient longitudinally, as this could introduce measurement error of 1–2°. Future research should integrate these validated non-weight-bearing assessments with dynamic, weight-bearing measures such as 3-D motion capture during gait and plantar pressure analysis. Although both methods demonstrate excellent reliability, using a consistent method ensures that the observed changes reflect true structural modifications rather than methodological differences. Future research should integrate these validated non-weight-bearing structural assessments with dynamic, weight-bearing evaluations such as 3-D motion capture during gait, plantar pressure distribution analysis, and functional movement assessments. Such multimodal approaches would provide a more comprehensive understanding of how structural forefoot alignment influences dynamic foot function and pathomechanics during weight-bearing activities.

The distinction between forefoot varus (a structural osseous alignment measured non-weight-bearing) and possible compensatory pronation (a functional adaptation measured weight-bearing, such as with the FPI) has important treatment implications.

Finally, when the longitudinal monitoring of forefoot alignment is clinically indicated, such as tracking deformity progression in a developing adolescent or evaluating the response to orthotic intervention, practitioners should maintain consistency in the measurement method throughout follow-up. Our findings indicate that although goniometry and radiography show strong agreement, method switching could introduce error of approximately 1–2°, which may obscure genuine clinical changes, particularly when those changes are subtle. Documentation of the specific measurement protocol used (including assessor positioning, ankle angle, and the landmark identification method) enhances the interpretability of serial measurements.

## 5. Conclusions

This study establishes marker-based radiographic measurement as a reliable reference standard for assessing shank–forefoot alignment (ICC = 0.906) and validates standardized clinical goniometry as an excellent alternative for routine assessment (ICC = 0.987). The strong agreement between methods (ICC = 0.898) supports the use of clinical goniometry for screening and longitudinal monitoring, reserving radiographic assessment for research contexts or cases requiring definitive structural confirmation. These findings provide the measurement foundation necessary for future research investigating the clinical significance of forefoot varus across the pathological spectrum and offer evidence-based guidance for practitioners selecting assessment methods appropriate to their clinical context.

## Figures and Tables

**Table 1:** Pearson correlation (ρ) among Mark 1, Mark 2, Nail 1 and Nail 2 measurements.

	Mark 1	Nail 1	Mark 2	Nail 2
Mark 1	1	0.919 (*p*-value ≈ 0)	0.591 (*p*-value ≈ 0)	0.607 (*p*-value ≈ 0)
Nail 1	0.919 (*p*-value ≈ 0)	1	0.591 (*p*-value ≈ 0)	0.645 (*p*-value ≈ 0)
Mark 2	0.591 (*p*-value ≈ 0)	0.591 (*p*-value ≈ 0)	1	0.911 (*p*-value ≈ 0)
Nail 2	0.607 (*p*-value ≈ 0)	0.645 (*p*-value ≈ 0)	0.911 (*p*-value ≈ 0)	1

**Table 2:** Intraclass Correlation Coefficients (ICCs) (two-way mixed effects, absolute agreement, and mean measures) among Mark 1, Mark 2, Nail 1 and Nail 2 for individual measurements.

	ICC(2,1)	IC 95%	SEM	MDC_95_	CV
Mark 1–Mark 2	0.589	[0.363; 0.750]	4.577	5.930	0.557
Nail 1–Nail 2	0.644	[0.439; 0.786]	4.376	5.798	5.102
Mark 1–Nail 1	0.915	[0.866; 0.946]	0.133	1.010	2.950
Mark 1–Nail 2	0.606	[0.385; 0.761]	0.005	0.192	0.051
Mark 2–Nail 1	0.582	[0.353; 0.745]	0.528	2.013	5.624
Mark 2–Nail 2	0.901	[0.828; 0.944]	0.024	0.433	0.515
Mark 1,2, Nail 1,2	0.707	[0.591; 0.807]	3.143	4.914	3.672

**Table 3:** Intraclass correlation coefficients (ICCs) among goniometry, Mark 1, Mark 2, Nail 1 and Nail 2 measurements.

Shank–Forefoot, Mark 1,2 and Nail 1,2	ICC(3,1)	IC95%	SEM	MDC_95_	CV
Individual measurements	0.639	[0.518; 0.753]	0.285	1.480	3.224839547
Mean measurements	0.898	[0.843; 0.938]	0.151	1.079	

**Table 4:** Inter-rater reliability coefficients for goniometric measurements.

Shank–Forefoot Researcher 1–Researcher 2	ICC(3,1)	IC 95%	SEM	MDC_95_	CV
Individual measurements	0.987	[0.977; 0.993]	0.017	0.364	0.974
Mean measurements	0.993	[0.988; 0.996]	0.013	0.312	

**Table 5:** Intraclass correlation coefficients (ICCs) among Mark 1, Mark 2, Nail 1 and Nail 2 with respect to shank–forefoot measurements for individual observations.

	ICC(3,1)	IC 95%	SEM	MDC_95_	CV
Mark 1—Shank–forefoot	0.488	[0.287; 0.648]	0.329	1.591	2.986
Mark 2—Shank–forefoot	0.585	[0.357; 0.747]	0.233	1.338	2.440
Nail 1—Shank–forefoot	0.542	[0.353; 0.688]	0.004	0.167	0.036
Nail 2—Shank–forefoot	0.58	[0.351; 0.743]	0.183	1.187	1.914

## Data Availability

The raw data supporting the conclusions of this article will be made available by the authors on request.

## Supplementary Materials

The following supporting information can be downloaded at: https://www.mdpi.com/article/10.3390/diagnostics16050703/s1, File S1: Supplementary Material FPI; File S2: Supplementary Material Mean Measurement and Correlation; File S3: Supplementary material Method; File S4: Supplementary Material Within-Participant Correlation.

## Abbreviations

BMI	Body Mass Index
CI	Confidence Interval
FPI	Foot Posture Index
FV	Forefoot Varus
GRRAS	Guidelines for Reporting Reliability and Agreement Studies
ICC	Intraclass Correlation Coefficient
IQR	Interquartile Range
MA	Meary’s Angle
MDC	Minimal Detectable Change
SD	Standard Deviation
SEM	Standard Error of Measurement
SFA	Shank–Forefoot Alignment
