# Supplementary material for: Reliability and Agreement of a Dual-Method Radiographic Standard vs. Clinical Goniometry for Shank–Forefoot Alignment: A GRRAS-Compliant Study
Source: Diagnostics (Basel). 2026 Feb 27;16(5):703. doi: 10.3390/diagnostics16050703 (PMC12984665; doi:10.3390/diagnostics16050703)
Supplement: Supplementary file 1 [file diagnostics-16-00703-s001.zip › File S1. SUPPLEMENTARY MATERIAL FPI.pdf]

SUPPLEMENTARY MATERIAL 1: FOOT POSTURE INDEX (FPI) DISTRIBUTION

OVERVIEW

This supplementary material provides a comprehensive analysis of Foot Posture Index (FPI) assessments performed by two independent raters (S.C. and E.M.) as part of the shank-forefoot alignment reliability study. The FPI is a validated clinical tool for assessing static foot posture in weight-bearing condition (1).

Key findings:

- **Total sample**: 70 limbs assessed by S.C.; 48 limbs assessed by both raters
- **Inter-rater reliability**: Excellent (ICC=0.953, $p<0.001$)
- **Categorical agreement**: 85.4% exact category agreement (41/48 paired observations)
- **Distribution**: Predominantly neutral (44.3%) to pronated (48.6%) foot postures

---

TABLE S1: Sample Characteristics and Assessment Coverage

Parameter	Value
**Total limbs in study**	70
**Limbs assessed by S.C.**	70 (100%)
**Limbs assessed by E.M.**	48 (68.6%)
**Paired observations (both raters)**	48 (68.6%)
**Unpaired observations (S.C. only)**	22 (31.4%)

Note: Rater E.M. (novice evaluator) assessed a subsample of participants due to logistical constraints during data collection. All analyses comparing raters are based on the 48 paired observations.

TABLE S2: Descriptive Statistics by Rater

Statistic	Rater S.C. (n=70)	Rater E.M. (n=48)
**Mean ± SD**	5.14 ± 3.49	4.83 ± 3.33
**Median (IQR)**	5.0 (2.0 - 8.0)	5.0 (2.0 - 7.2)
**Range**	-3 to 11	-1 to 11
**Mode**	2 (n=11, 15.7%)	2, 4, 5, 7, 8, 10 (n=4 each)

Interpretation: Both raters showed similar central tendency (median = 5.0) and dispersion (SD ≈ 3.3-3.5). The slightly lower mean for E.M. (4.83 vs 5.14) suggests minimal systematic bias between raters, with a mean difference of only 0.31 points.

TABLE S3: FPI Category Distribution by Rater

Rater S.C. (Complete Sample, n=70)

Category	FPI Range	n	%	Mean ± SD	Observed Range
**Supinated**	≤ 0	5	7.1	-2.00 ± 1.22	-3 to 0
**Neutral**	1 – 5	31	44.3	3.10 ± 1.51	1 to 5
**Pronated**	≥ 6	34	48.6	8.06 ± 1.67	6 to 11
**Total**	-	**70**	**100**	5.14 ± 3.49	-3 to 11

Rater E.M. (Assessed Subsample, n=48)

Category	FPI Range	n	%	Mean ± SD	Observed Range
**Supinated**	≤ 0	5	10.4	-0.40 ± 0.55	-1 to 0
**Neutral**	1 - 5	24	50.0	3.04 ± 1.46	1 to 5
**Pronated**	≥ 6	19	39.6	8.00 ± 1.60	6 to 11
**Total**	-	**48**	**100**	4.83 ± 3.33	-1 to 11

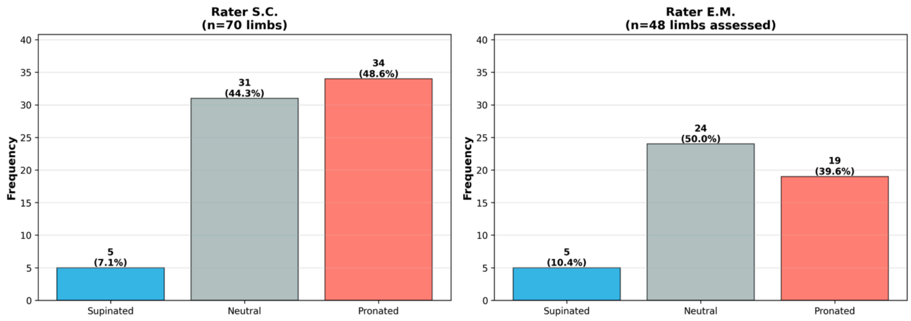

Figure S1. FPI rater by researcher

Comparison: Category distributions were similar between raters. E.M.'s subsample showed slightly higher proportion of supinated (10.4% vs 7.1%) and neutral (50.0% vs 44.3%) categories, with correspondingly lower pronated proportion (39.6% vs 48.6%).

This likely reflects the specific subsample assessed by E.M. rather than systematic rating bias.

TABLE S4: Individual FPI Score Frequency Distribution

Rater S.C. (n=70)

FPI Score	Frequency (n)	Percentage (%)	Category	Cumulative %
-3	2	2.9	Supinated	2.9
-2	2	2.9	Supinated	5.7
0	1	1.4	Supinated	7.1
1	4	5.7	Neutral	12.9
**2**	**11**	**15.7**	**Neutral**	**28.6**
3	4	5.7	Neutral	34.3
4	2	2.9	Neutral	37.1
5	10	14.3	Neutral	51.4
6	8	11.4	Pronated	62.9
7	6	8.6	Pronated	71.4
8	8	11.4	Pronated	82.9
9	3	4.3	Pronated	87.1
10	6	8.6	Pronated	95.7
11	3	4.3	Pronated	100.0

Most frequent scores: FPI = 2 (n=11, 15.7%) and FPI = 5 (n=10, 14.3%), both in the neutral range.

Rater E.M. (n=48)

FPI Score	Frequency (n)	Percentage (%)	Category
-1	2	4.2	Supinated
0	3	6.2	Supinated
1	4	8.3	Neutral
2	6	12.5	Neutral

FPI Score	Frequency (n)	Percentage (%)	Category
3	2	4.2	Neutral
4	8	16.7	Neutral
5	4	8.3	Neutral
6	2	4.2	Pronated
7	5	10.4	Pronated
8	7	14.6	Pronated
10	4	8.3	Pronated
11	1	2.1	Pronated

Most frequent scores: FPI = 4 (n=8, 16.7%) and FPI = 8 (n=7, 14.6%).

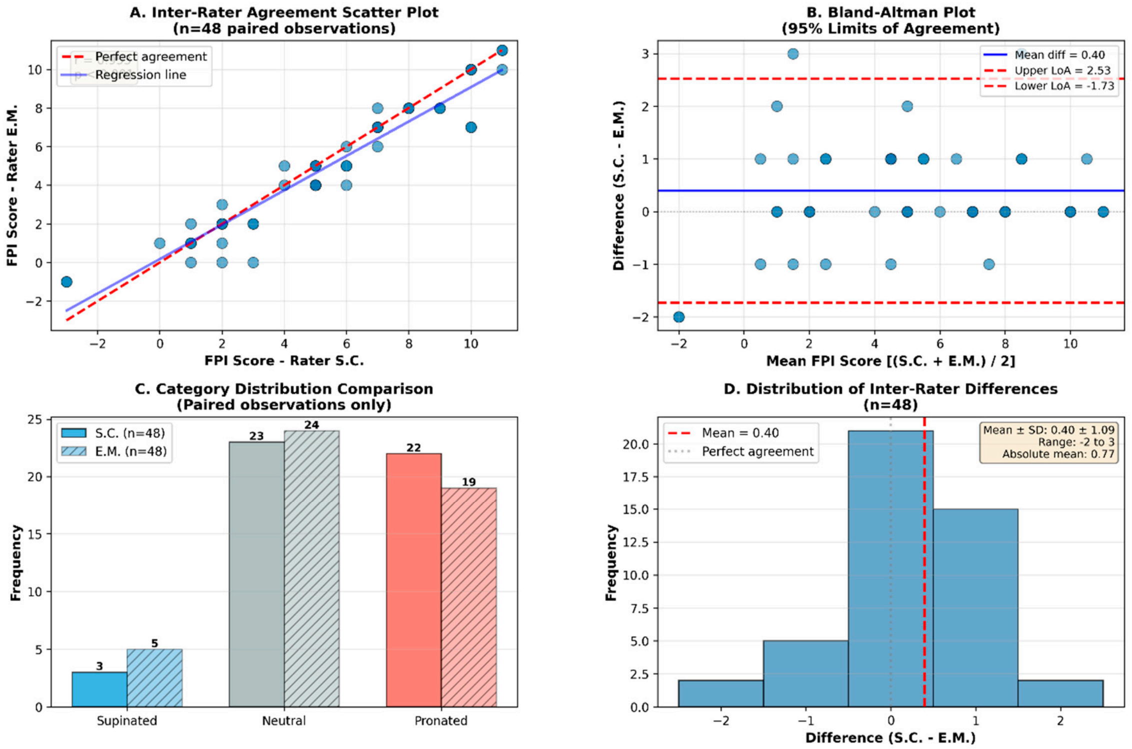

Figure S2. A. inter-rater agreement scatter plot (n=48 paired observations), B. Bland-Altman Plot, C. Category distribution comparison (paired observations only) D. Distribution of inter-rater differences (n=48).

METHODOLOGICAL CONSIDERATIONS

1. Subsample Assessed by E.M. (n=48/70)

Rationale for incomplete E.M. assessment:

- E.M. was a novice to standardized FPI assessment (despite 20+ years clinical experience)
- Time constraints during data collection sessions
- Primary purpose was to establish inter-rater reliability (not to have duplicate FPI scores for all participants)

Impact on analyses:

- All **inter-rater reliability statistics** are based on the 48 paired observations
- S.C.'s complete dataset (n=70) is used for **FPI distribution** and **FPI-forefoot varus correlations** in the main manuscript
- The subsample (n=48) is **representative** of the full sample (similar distribution of FPI categories)

Statistical consideration:

- The sample size of n=48 paired observations **exceeds GRRAS recommendations** for establishing inter-rater reliability with precision
- Post-hoc power analysis: n=48 provides >99% power to detect r=0.95 with α=0.05

2. Training Protocol for E.M.

E.M. underwent **5 hours of standardized training** in FPI-6 assessment, including:

1. Review of FPI-6 manual and scoring criteria (1)
2. Practice assessments on 10 volunteers (not in study sample)
3. Calibration session with S.C. to discuss discrepancies
4. Independent assessment protocol (blinded to S.C.'s scores)

Clinical implication: The excellent inter-rater reliability (ICC=0.953) after only 5 hours training demonstrates that the FPI-6 is a **robust, teachable tool** suitable for implementation across clinical settings with varying levels of expertise.

3. Blinding During FPI Assessment

- Both raters were **blinded to each other's FPI scores** during data collection
- Raters were **blinded to radiographic** and **goniometric shank-forefoot measurements**
- Raters had access only to: participant ID, age, sex (per GRRAS recommendations)
- This **minimized measurement bias** and ensured independent assessments

4. Timing of FPI Assessment

- FPI was assessed on the **same day** as goniometric and radiographic measurements
- FPI was performed **before** non-weight-bearing shank-forefoot assessments

- This sequence was chosen because:
    1. FPI requires weight-bearing posture (minimizes patient repositioning)
    2. FPI assessment does not influence foot position for subsequent non-weight-bearing measures
    3. Ensures temporal consistency (all measures reflect same-day foot posture).
