# Supplementary material for: Reliability and Agreement of a Dual-Method Radiographic Standard vs. Clinical Goniometry for Shank–Forefoot Alignment: A GRRAS-Compliant Study
Source: Diagnostics (Basel). 2026 Feb 27;16(5):703. doi: 10.3390/diagnostics16050703 (PMC12984665; doi:10.3390/diagnostics16050703)
Supplement: Supplementary file 1 [file diagnostics-16-00703-s001.zip › File S2. supplementary material mean measure and correlation.pdf]

	ICC(2,1)	IC95%	SEM	MDC95
Mark 1 – Mark 2	0,742	[0.533; 0.857]	0,042	0,571
Nail 1 – Nail 2	0,784	[0.609; 0.880]	0,343	1,623
Mark 1 – Nail 1	0,956	[0.928; 972]	0,095	0,856
Mark 1 – Nail 2	0,754	[0.556; 0.864]	0,004	0,171
Mark 2 – Nail 1	0,736	[0.522; 0.854]	0,419	1,795
Mark 2 – Nail 2	0,948	[0.906; 0.971]	0,018	0,368
Mark 1,2, Nail 1,2	0,906	[0.852; 0.944]	0,166	1,129

Table S1. ICC = Intraclass Correlation Coefficient (two-way mixed effects, absolute agreement, mean measures) among Mark 1, Mark 2, Nail 1 and Nail 2 for mean measures.

	Mark 1	Nail 1	Mark 2	Nail 2
FPI Researcher 1	0.093 (p-value=0.443)	0.208 (p-value=0.084)	0.341 (p-value=0.02)	0.430 (p-value=0.003)

Table S2. Pearson correlation (ρ) among Mark 1, Mark 2, Nail 1, Nail 2 and FPI measure taken by researcher 1.

Nail 2 presented high positive correlation with FPI measures by S.C. (researcher 1 in Table S2). BMI didn't present significant correlation with FPI measurements taken by S.C. ($\rho=0.035$; p-value=0.774)

FPI Researcher 1 - Researcher	ICC(3,1)	IC95%	SEM	MDC95	CV
Individual measures	0,951	[0.913; 0.972]	0,062	0,689	5,551
Mean measures	0,975	[0.955; 0.986]	0,044	0,582	

Table S3. intraclass correlation coefficients (ICC) among FPI measures

	Mark 1	Nail 1	Mark 2	Nail 2
Goniometry	0.919 (p-value≈0)	0.548 (p-value≈0)	0.585 (p-value≈0)	0.583 (p-value≈0)

Table S4. Pearson correlation (ρ) among Mark 1, Mark 2, Nail 1, Nail 2 and shank forefoot measure.

Marks 1, 2 and nails 1 and 2 presented high positive and significant correlations (Table S4).

	ICC(3,1)	IC95%	SEM	MDC95
Mark 1 – Shank forefoot	0,656	[0.446; 0.786]	0,270	1,440
Mark 2 – Shank forefoot	0,738	[0.526; 0.855]	0,185	1,193
Nail 1 - Shank forefoot	0,703	[0.522; 0.815]	0,003	0,150
Nail 2 – Shank forefoot	0,734	[0.519; 0.853]	0,146	1,059

Table S5. intraclass correlation coefficients (ICC) among Mark 1, Mark 2, Nail 1 and Nail 2 respect to shank forefoot for mean measures

Marks 1, 2 and nails 1 and 2 presented high positive and significant correlations (see Table S6). ICC among first and second shank forefoot measures indicates good reliability for individual and excellent reliability for mean measures (Table S7).

	Mark 1	Nail 1	Mark 2	Nail 2
Shank forefoot second measure	0.489 (p-value≈0)	0.554 (p-value≈0)	0.724 (p-value≈0)	0.748 (p-value≈0)

Table S6. Pearson correlation (ρ) among Mark 1, Mark 2, Nail 1, Nail 2 and shank forefoot measure.

Shank forefoot 1,2	ICC(2,1)	IC95%	SEM	MDC95
Single rater (mean of 3 measurements)	0.857	[0.756; 0.918]	0.077	0.772
Mean measures	0.923	[0.861; 0.957]	3.545	5.219

Table S7. intraclass correlation coefficients (ICC) among shank 1 and 2 measures

Descriptive Statistics - Paired Subsample (n=48): Mean SFA values were nearly identical

between raters:

- S.C.: 15.47±7.68° (range: -1.00° to 29.66°)
- E.M.: 15.68±7.48° (range: -0.33° to 32.00°)

The mean difference (S.C. - E.M.) was -0.21±1.22°, indicating negligible systematic bias between raters. The mean absolute difference was 0.98°, with 70.8% of paired measurements differing by ≤1° and 89.6% by ≤2°.

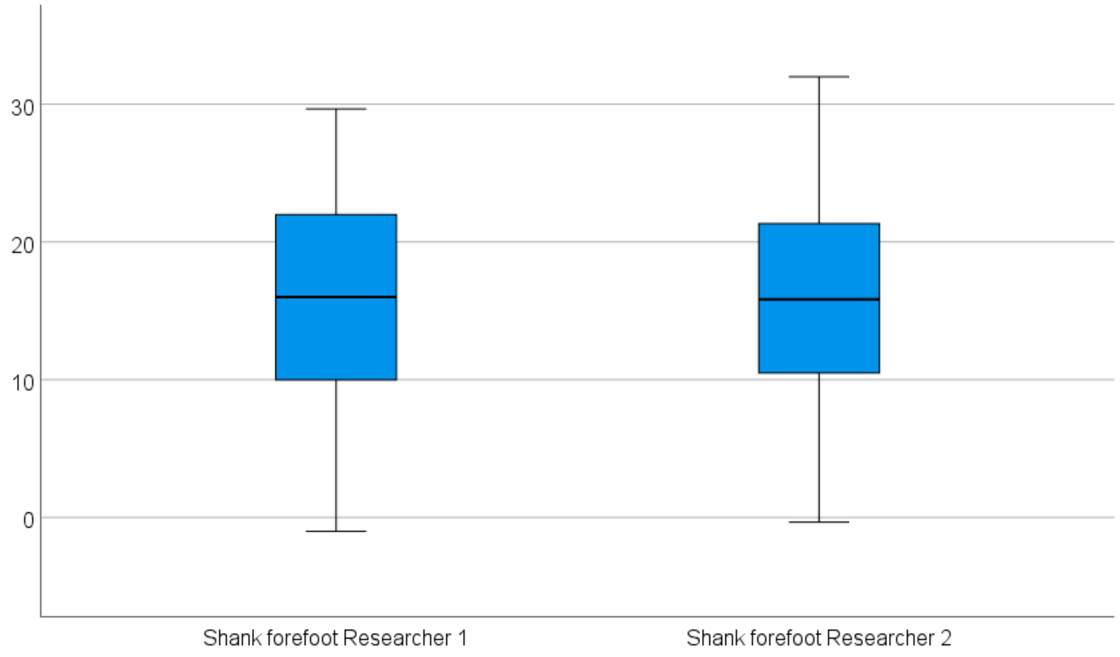

Figure S1: Boxplot for shank forefoot measures by researcher.

Figure S2 presents the boxplot for shank forefoot second measure by FPI.

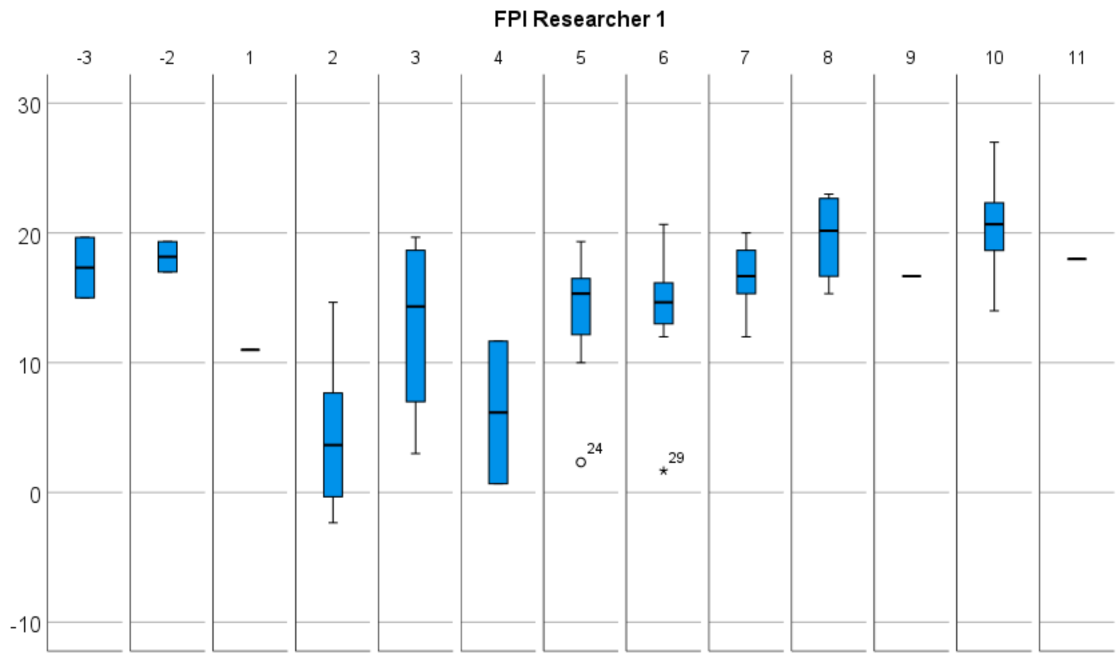

Figure S2: Boxplot for shank forefoot measures by FPI.
