# Supplementary material for: Reliability and Agreement of a Dual-Method Radiographic Standard vs. Clinical Goniometry for Shank–Forefoot Alignment: A GRRAS-Compliant Study
Source: Diagnostics (Basel). 2026 Feb 27;16(5):703. doi: 10.3390/diagnostics16050703 (PMC12984665; doi:10.3390/diagnostics16050703)
Supplement: Supplementary file 1 [file diagnostics-16-00703-s001.zip › File S3. Supplementary material Method.pdf]

SUPPLEMENTARY MATERIAL - DETAILED METHODOLOGY

Study Title: Reliability and Agreement of a Dual-Method Radiographic Standard vs. Clinical Goniometry for Shank-Forefoot Alignment: A GRRAS-Compliant Study

---

TABLE OF CONTENTS

1. Detailed Assessor Characteristics and Training Protocol
2. Sample Size Calculations
3. Detailed Recruitment and Sampling Procedures
4. Detailed Measurement Protocols
5. Equipment Specifications
6. GRRAS Checklist Compliance
7. References

---

1. DETAILED ASSESSOR CHARACTERISTICS AND TRAINING PROTOCOL

1.1 Assessor Profiles

Assessor 1: S.C. (Goniometric Evaluator - Expert)

- **Professional qualification:** Licensed Physical Therapist
- **Clinical experience:** +15 years of clinical practice in biomechanical assessment
- **Specialization:** Lower extremity biomechanics and foot alignment assessment
- **Previous experience with protocol:** Extensive experience with standardized goniometric forefoot assessment protocols, including the Mendonça protocol
- **Role in study:** Primary goniometric evaluator for inter-rater and intra-rater reliability assessments
- **Language:** Native Spanish speaker
- **Institutional affiliation:** Departmental Section of Podiatry, University of Barcelona

Assessor 2: E.M. (Goniometric Evaluator - Novice)

- **Professional qualification:** Podiatric Surgeon

- **Clinical experience:** 20+ years of clinical experience in podiatric surgery and foot deformity correction
- **Specialization:** Surgical management of foot and ankle pathologies
- **Previous experience with protocol:** Extensive clinical experience in foot assessment but no prior experience with the standardized Mendonça goniometric protocol before study training
- **Role in study:** Secondary goniometric evaluator for inter-rater reliability assessment
- **Training received:** Completed 5-hour standardized training protocol before data collection
- **Language:** Native Spanish speaker
- **Institutional affiliation:** Departmental Section of Podiatry, University of Barcelona

Assessor 3: V.O. (Radiographic Evaluator)

- **Professional qualification:** Board-certified Radiologist
- **Clinical experience:** 15+ years of experience in musculoskeletal imaging and diagnostic radiology
- **Specialization:** Digital radiographic measurements of skeletal alignment and foot-ankle complex imaging
- **Previous experience:** Routine clinical experience in digital radiographic measurements using specialized software for angle calculations
- **Role in study:** Sole radiographic evaluator for all radiographic measurements (both inter-rater and intra-rater reliability assessments)
- **Language:** Native Spanish speaker
- **Institutional affiliation:** Hospital Podològic Universitat de Barcelona

Statistical Analyst: C.S.

- **Professional qualification:** Statistician and Computer Scientist
- **Specialization:** Biostatistics and reliability studies
- **Role in study:** Independent statistical analyst blinded to evaluator identity and measurement outcomes

- **Responsibility:** All data processing, statistical analyses, and interpretation of reliability coefficients
- **Institutional affiliation:** Computer Science and Statistics Department, Rey Juan Carlos University

---

1.2 Training Protocol

Overview

A standardized 5-hour training protocol was implemented for both goniometric evaluators (S.C. and E.M.) before initiating data collection. The training was designed to ensure measurement consistency and protocol adherence, divided into 2 sessions conducted over two consecutive days.

Training Objectives

- Standardize patient positioning and landmark identification
- Ensure consistent goniometer placement and angle reading
- Minimize inter-rater variability through protocol standardization
- Establish competency criteria before study initiation
- Practice blinding procedures and data recording

SESSION 1: Theoretical Foundation and Practical Demonstration (2.5 hours)

Part A: Theoretical Review (1 hour)

- Review of forefoot varus anatomy and biomechanics
- Detailed review of the Mendonça et al. (2013) shank-forefoot alignment measurement protocol
- Review of Foot Posture Index (FPI-6) criteria and scoring system (Redmond et al., 2006)
- Discussion of common measurement errors identified in reliability literature:
    - Inconsistent ankle positioning (Van Gheluwe et al., 2002)
    - Variable landmark identification (Elveru et al., 1988)

    - Goniometer misalignment (Picciano et al., 1993)
- Review of study protocol, blinding procedures, and GRRAS guidelines (Kottner et al., 2011)
- Review of ethical considerations and radiation safety protocols

Part B: Practical Demonstration (1.5 hours)

Shank-forefoot alignment goniometry (1 hour):

- Step-by-step demonstration by expert evaluator (S.C.) on volunteer participants
- Correct patient positioning: prone with knees extended, ankle at 90° dorsiflexion
- Anatomical landmark identification:
    - Tibial bisector (midpoint between malleoli through calf midline)
    - Metatarsal heads (1st and 5th)
    - Proper palpation techniques to ensure accurate landmark location
- Metallic nail placement under metatarsal heads:
    - Horizontal positioning perpendicular to long axis of foot
    - Simultaneous contact with both 1st and 5th metatarsal heads
    - Verification of proper placement through palpation
- Goniometer placement and alignment:
    - Axis placement at ankle midpoint (between malleoli)
    - Stationary arm aligned with tibial bisector
    - Mobile arm aligned with metallic nail
    - Verification of frontal plane alignment
- Angle reading technique (to nearest degree)
- Three-measurement protocol with complete repositioning between measurements

Foot Posture Index assessment (0.5 hours):

- Demonstration of correct patient positioning (relaxed standing)
- Step-by-step demonstration of each of the 6 FPI criteria:

1. Talar head palpation (medial and lateral)
2. Curves above and below lateral malleolus
3. Calcaneal frontal plane position (inversion/eversion)
4. Bulge in talonavicular joint region
5. Medial longitudinal arch congruence
6. Forefoot-to-rearfoot alignment (abduction/adduction)

- Scoring system: -2 (supinated) to +2 (pronated) for each criterion
- Practice of borderline scoring (e.g., distinguishing between +1 and +2)
- Common errors and how to avoid them

SESSION 2: Supervised Practice and Competency Assessment (2.5 hours)

Part A: Supervised Practice (1.5 hours)

- Hands-on practice with 5 volunteer participants (not included in final study sample)
- Volunteers selected to represent range of forefoot alignments (varus, neutral, valgus)

Shank-forefoot alignment practice (1 hour):

- Each trainee performed complete measurement protocol on practice volunteers
- Immediate feedback provided by expert evaluator (S.C.) on:
    - Patient positioning accuracy
    - Landmark identification precision
    - Nail placement correctness
    - Goniometer alignment
    - Angle reading accuracy
- Real-time correction of errors
- Practice of three consecutive measurements per subject
- Practice of complete goniometer repositioning between measurements
- Discussion of measurement variability sources

FPI assessment practice (0.5 hours):

- Independent FPI scoring on practice subjects
- Immediate comparison with expert evaluator (S.C.) scoring
- Discussion of scoring discrepancies, particularly for:
    - Borderline criteria (e.g., distinguishing 0 vs +1)
    - Criteria requiring subjective judgment (e.g., arch congruence)
- Additional practice on subjects with ambiguous presentations
- Practice of systematic evaluation approach (same order every time)

Part B: Competency Assessment (1 hour)

- Independent measurements on 3 additional volunteers
- Both shank-forefoot alignment and FPI assessed on each volunteer
- Measurements performed simultaneously by trainee (E.M.) and expert (S.C.)
- Measurements conducted under simulated study conditions:
    - Blinded to each other's results
    - No communication during assessment
    - 30-minute interval between evaluators
- Calculation of agreement between trainee and expert for both measures
- Discussion of any persistent discrepancies
- Additional focused practice if competency criteria not met on first attempt

---

1.3 Competency Criteria

Evaluators were considered competent when all of the following criteria were achieved:

For Shank-Forefoot Alignment:

- Intraclass correlation coefficient (ICC) >0.85 with expert evaluator (S.C.) on practice sample
- Consistent and accurate patient positioning verified by expert observation (100% of attempts)

- Correct anatomical landmark identification in >95% of attempts
- Appropriate goniometer placement verified by expert (>95% of attempts)
- Angle readings within ±2° of expert evaluator in >90% of measurements
- Completion of measurement protocol within expected time frame (15-20 minutes per participant for bilateral assessment)

For FPI Assessment:

- Agreement within 1 point on each individual FPI criterion for >90% of assessments
- ICC >0.85 for total FPI scores with expert evaluator
- Correct identification and palpation of anatomical landmarks (100% of attempts)
- Systematic evaluation approach maintained across all assessments
- Total FPI classification (supinated/normal/pronated) agreement >90%

General Competency Requirements:

- Demonstrated understanding of blinding procedures
- Accurate and complete data recording
- Adherence to standardized protocol without prompting
- Professional interaction with study participants

1.4 Training Results

Both evaluators (S.C. and E.M.) successfully met all competency criteria at the end of the 5-hour training protocol:

Shank-Forefoot Alignment:

- ICC between S.C. and E.M. during training: 0.89 (95% CI: 0.78-0.95)
- Mean difference: 1.2° (SD: 2.1°)
- Agreement within ±2°: 94% of measurements

FPI Total Score:

- ICC between S.C. and E.M. during training: 0.91 (95% CI: 0.82-0.96)
- Perfect agreement (same score): 67% of assessments

- Agreement within ±1 point: 94% of assessments
- Classification agreement (supinated/normal/pronated): 100%

No additional training was required beyond the planned 5-hour protocol.

---

2. SAMPLE SIZE CALCULATIONS

2.1 Theoretical Framework

Sample size calculations for reliability studies were based on the approach described by Walter et al. (1998), which provides formulas for determining the number of subjects needed to achieve desired precision in intraclass correlation coefficient (ICC) estimation.

2.2 Inter-Rater Reliability Sample Size

Objective: Determine the minimum sample size needed for precise estimation of ICC with 95% confidence interval width of ±0.15.

Method: Walter et al. (1998) formula for ICC estimation in reliability studies.

Parameters:

- Minimally acceptable ICC (ρ_0): 0.65
    - Rationale: Based on literature review showing that ICC <0.65 is generally considered inadequate for clinical measurements (Kottner and Dassen, 2008)
- Expected ICC (ρ_1): 0.85
    - Rationale: Based on pilot data and previous literature on photogrammetric shank-forefoot alignment methods (Mendonça et al., 2013; Diniz et al., 2020)
- Number of raters (k): 2
- Desired 95% confidence interval width: ±0.15
    - Rationale: Width of 0.30 (±0.15) provides clinically meaningful precision for reliability estimation (Shrout and Fleiss, 1979)
- Significance level (α): 0.005
    - Rationale: More stringent threshold to reduce Type I error probability from ~26% to ~1% (Colquhoun, 2014)
- Statistical power ($1-\beta$): 80%

Calculation: Using the Walter et al. (1998) formula:

$n = 1 + [Z_{1-\alpha/2} \times \sqrt{(1-\rho_1)}/(\rho_1 \times \sqrt{(k-1)})]^2$

Where:

- $Z_{1-\alpha/2} = 2.807$ for $\alpha = 0.005$ (99.5% confidence)
- $\rho_1 = 0.85$ (expected ICC)
- $k = 2$ (number of raters)

Result:

- Minimum required sample size: 73 limbs
- Accounting for potential measurement unit (limbs): n = 37 participants × 2 limbs = 74 limbs

Actual recruitment:

- Achieved: 35 participants (70 limbs)
- Rationale for slight under-recruitment:
  - 70 limbs still provides 96% of planned statistical power
  - Adequate precision maintained for primary analyses (95% CI width increases from ±0.15 to ±0.16)
  - Recruitment window limited by ethical approval period and resource constraints

---

2.3 Intra-Rater Reliability Sample Size

Objective: Determine appropriate subsample size for intra-rater reliability with sufficient precision.

Method: GRRAS recommendations (Kottner et al., 2011) combined with Walter et al. (1998) approach for test-retest designs.

Parameters:

- Expected ICC for intra-rater reliability: 0.90
  - Rationale: Intra-rater reliability typically exceeds inter-rater reliability by 0.05-0.10 (Streiner and Norman, 2008)

- Number of measurements per subject: 2 (test-retest)
- Desired 95% CI width: ±0.15
- Significance level (α): 0.005
- Statistical power (1-β): 80%

Calculation: Using modified Walter et al. (1998) formula for repeated measurements:

$n = 1 + [Z_{1-\alpha/2} \times \sqrt{(2(1-\rho)^2[1+(k-1)\rho]^2)/(k(k-1)\rho^2)}]$

Where:

- $\rho = 0.90$ (expected intra-rater ICC)
- $k = 2$ (test-retest measurements)

Result:

- Minimum required sample size: 20 participants (40 limbs)

Actual recruitment:

- Achieved: 23 participants (46 limbs)
- Rationale for oversampling:
    - Buffer against potential loss to follow-up (anticipated 10-15% attrition)
    - Ensures precision even with complete data (actual attrition: 0%)
    - Provides adequate power for exploratory subgroup analyses

---

2.4 Justification for Bilateral Measurements

Methodological Approach: Both limbs were measured in each participant to increase statistical power while acknowledging potential within-subject correlation.

Justification:

- Widely accepted practice in foot morphology reliability studies (Menz et al., 2013)
- Increases effective sample size without proportional increase in recruitment burden
- Appropriate for studies focused on measurement precision rather than population prevalence (Kottner et al., 2011)

Statistical Considerations:

- Potential within-subject correlation addressed through:
    - Mixed-effects models accounting for clustering (Shrout and Fleiss, 1979)
    - Sensitivity analyses comparing results with and without bilateral data
    - Assessment of laterality effects (no significant differences found: T=0.22, p=0.827)

Precedent in Literature:

- Consistent with GRRAS guidelines for reliability studies (Kottner et al., 2011)
- Supported by methodological papers on reliability research design (Shoukri, 2004)
- Used in previous forefoot alignment reliability studies (Mendonça et al., 2013; Diniz et al., 2020)

2.5 Post-Hoc Power Analysis

Inter-Rater Reliability (n=70 limbs):

- Achieved power: 96% for detecting ICC ≥0.85
- 95% CI width achieved: ±0.16 (target: ±0.15)
- Conclusion: Adequate power maintained despite slight under-recruitment

Intra-Rater Reliability (n=46 limbs):

- Achieved power: 91% for detecting ICC ≥0.90
- 95% CI width achieved: ±0.14 (target: ±0.15)
- Conclusion: Exceeded target precision due to oversampling

---

3. DETAILED RECRUITMENT AND SAMPLING PROCEDURES

3.1 Recruitment Setting

Institution: Hospital Podològic Universitat de Barcelona

- **Management:** Fundació Josep Finestres
- **Location:** Barcelona, Catalonia, Spain
- **Type:** University-affiliated podiatric specialty hospital

- **Patient population:** General adult population seeking podiatric care or assessment
- **Recruitment period:** January 1, 2024 to May 31, 2024 (5 months)

3.2 Consecutive Sampling Protocol

Rationale: Consecutive sampling was chosen to:

- Enhance ecological validity by including representative clinical population
- Minimize selection bias inherent in convenience sampling
- Ensure temporal distribution of recruitment across study period
- Maintain feasibility within resource constraints

Detailed Procedure:

1. **Screening Phase:**
    - Every patient attending Hospital Podològic during study period was screened for eligibility
    - Screening conducted by research team member (S.C.) during initial clinic visit
    - Screening log maintained to document all approached patients
2. **Eligibility Assessment:**
    - Inclusion criteria verified through:
        - Age verification (government-issued ID)
        - Clinical assessment of ankle dorsiflexion capability (functional test)
        - Verbal confirmation of willingness to participate in both protocols
    - Exclusion criteria assessed through:
        - Medical history review (previous surgeries, neurological conditions)
        - Clinical examination (acute injury, inflammation)
        - Pregnancy screening (self-report for females of childbearing age)
3. **Invitation and Consent:**
    - Eligible patients were invited to participate in order of clinic arrival (consecutive approach)
    - Study information sheet provided in Spanish

- Verbal explanation of study procedures, risks, and benefits
- Written informed consent obtained from all participants who agreed
- Participants allowed minimum 24 hours to consider participation (for non-urgent assessments)

4. **Scheduling:**
    - Participants scheduled for measurement session within 2 weeks of enrollment
    - Flexible scheduling offered to maximize participation
    - Reminder phone call 2 days before scheduled session

3.3 Screening and Enrollment Results

Screening Flow:

- Total patients attending Hospital Podològic during study period: 127
- Patients meeting initial age criterion (≥18 years): 98 (77.2%)
- Patients meeting all inclusion criteria: 52 (40.9% of total screened)
- Patients approached and invited to participate: 52 (100% of eligible)

Enrollment Outcomes:

- Patients agreeing to participate: 35 (67.3% acceptance rate)
- Patients declining participation: 17 (32.7%)
- Patients enrolled and completing baseline assessment: 35 (100% retention at baseline)
- Dropouts after enrollment: 0 (0% attrition)

Reasons for Declining Participation (n=17):

- Time constraints / scheduling conflicts: 12 (70.6%)
    - Unable to attend follow-up session
    - Work schedule incompatibility
- Lack of interest / no perceived benefit: 3 (17.6%)
- Concerns about radiation exposure: 2 (11.8%)

- Other / not specified: 0 (0%)

Reasons for Ineligibility (n=46):

- Age <18 years: 29 (63.0%)
- Inability to maintain ankle dorsiflexion at 90°: 8 (17.4%)
  - Fatigue holding ankle position: 5
  - Previous ankle injury/surgery: 3
- History of major lower limb surgery: 4 (8.7%)
- Acute foot/ankle injury or inflammation: 3 (6.5%)
- Neurological condition affecting foot: 1 (2.2%)
- Pregnancy: 1 (2.2%)

3.4 Comparison of Participants vs. Non-Participants

Available Demographic Data: Due to ethical constraints, limited demographic data were available for patients who declined participation. Comparison based on available clinic records:

Age:

- Participants (n=35): Mean 32.7±10.8 years (range: 19-67)
- Non-participants (n=17): Estimated mean 34.2 years (based on clinic records)
- No systematic age differences observed (informal assessment)

Sex:

- Participants: 54% female
- Non-participants: Approximately 50% female (based on clinic records)
- No systematic sex differences observed

Note: Formal statistical comparison was not conducted due to:

- Ethical limitations on data collection from non-participants
- Limited availability of demographic data for declined patients
- Small sample size of non-participants

3.5 Detailed Inclusion and Exclusion Criteria

Inclusion Criteria (with rationale):

1. **Age ≥18 years**
   - Rationale: Ensure informed consent capacity; foot structure maturation complete
   - Verification method: Government-issued identification document
2. **Ability to actively maintain ankle dorsiflexion at 90°**
   - Rationale: Required for standardized measurement position per Mendonça protocol
   - Verification method: Clinical functional test (patient actively dorsiflexes ankle to 90° and maintains for 30 seconds without discomfort)
3. **Willingness to participate in both goniometric and radiographic protocols**
   - Rationale: Complete data necessary for method comparison
   - Verification method: Verbal confirmation during consent process
4. **Ability to understand and provide informed consent**
   - Rationale: Ethical requirement
   - Verification method: Comprehension assessment through teach-back method

Exclusion Criteria (with rationale):

1. **History of major lower limb surgery affecting foot structure**
   - Rationale: Surgical alteration may compromise validity of alignment measurements
   - Examples: Osteotomies, arthrodesis, joint replacement
   - Verification: Medical history review and clinic records
   - Note: Minor procedures (e.g., nail surgery, soft tissue procedures) were NOT exclusionary
2. **Neurological conditions affecting foot posture or muscle control**

- Rationale: Neuromuscular disorders may introduce measurement variability unrelated to structural alignment
- Examples: Peripheral neuropathy with motor deficit, cerebral palsy, stroke with residual hemiparesis
- Verification: Medical history and neurological screening examination
- Note: Sensory neuropathy without motor involvement was NOT exclusionary

3. **Acute injury or inflammation of foot or ankle at time of assessment**
    - Rationale: Acute conditions may temporarily alter foot posture; pain may limit positioning
    - Examples: Acute ankle sprain, plantar fasciitis flare, gout attack
    - Verification: Clinical examination and patient report
    - Note: Chronic stable conditions (e.g., resolved previous injuries) were NOT exclusionary
4. **Pregnancy**
    - Rationale: Radiation exposure contraindicated; physiological foot changes during pregnancy
    - Verification: Self-report for females of childbearing age
5. **Inability to tolerate prone positioning for measurement procedures**
    - Rationale: Required body position for standardized measurement
    - Examples: Severe respiratory condition, claustrophobia in prone position
    - Verification: Patient report and physical tolerance test

---

3.6 Follow-Up for Intra-Rater Reliability

Subsample Selection:

- **Method:** First 23 participants who agreed to return for follow-up assessment
- **No selection based on:**
    - Measurement results (degree of forefoot varus)

    - Demographic characteristics
    - Foot pathology status
- **Rationale:** Minimize selection bias; representative subsample of main cohort

Follow-Up Protocol:

- **Minimum washout period:** more than 30 days
    - Rationale: Sufficient time to minimize recall bias while maintaining structural stability (Kottner et al., 2011)
- **Scheduling considerations:**
    - Same time of day as initial assessment when possible (±2 hours)
    - Same day of week when feasible to control for potential weekly activity patterns
    - Participants instructed to maintain usual footwear and activity patterns

Contact and Retention Procedures:

- Participants contacted by phone 1 week before scheduled follow-up
- Reminder phone call 2 days before appointment
- Flexible rescheduling offered if participant unable to attend original appointment
- No financial incentive provided (participation voluntary)

Follow-Up Results:

- Participants initially agreeing to follow-up: 23 (100% of those invited; 65.7% of total sample)
- Participants completing follow-up assessment: 23 (100% retention rate)
- No adverse events or complications reported
- No significant changes in foot health status between assessments (per participant report)

4. DETAILED MEASUREMENT PROTOCOLS

4.1 Assessment Order and Session Structure

Rationale for Assessment Order: The order of assessments was standardized to minimize influence of one measurement on another and to maintain consistency with standard clinical practice.

Standardized Order for All Participants:

1. **FPI assessment** (standing) - performed FIRST
2. **Shank-forefoot alignment goniometry** (prone) - performed SECOND
3. **Radiographic assessments** (prone) - performed LAST

Justification:

- FPI first (standing) to avoid any influence from prone positioning on foot posture
- Goniometry before radiography to allow familiarization with prone position
- Radiography last to minimize radiation exposure duration and allow patient to rest between goniometric measurements

Session Timeline:

- Total session duration: Approximately 60 minutes
- FPI assessment: 10 minutes (bilateral)
- 5-minute rest/position change
- Goniometric assessment by Evaluator 1: 20 minutes (bilateral, 3 measurements per foot)
- 30-minute interval (participant rests in waiting area)
- Goniometric assessment by Evaluator 2: 20 minutes (bilateral, 3 measurements per foot)
- 5-minute rest/position change
- Radiographic assessment: 15 minutes (bilateral, both methods)

4.2 Foot Posture Index (FPI-6) Assessment Protocol

4.2.1 Background and Rationale

The FPI-6 is a validated observational clinical assessment tool that quantifies standing foot posture through six specific criteria (Redmond et al., 2006). It has demonstrated:

- Excellent inter-rater reliability: ICC 0.91-0.93 (Redmond et al., 2006)

- Excellent intra-rater reliability: ICC 0.87-0.94 (Evans et al., 2003)
- Good correlation with radiographic and 3D measures (Redmond et al., 2008)

Purpose in Current Study:

- Exploratory assessment of relationship between structural forefoot alignment (shank-forefoot angle) and functional foot posture (FPI)
- Characterize sample in terms of static foot posture distribution
- Provide context for interpreting forefoot varus measurements

4.2.2 Equipment

- **None required** (purely observational tool based on visual inspection and manual palpation)
- **Materials used:**
    - FPI-6 data recording sheet (Spanish version)
    - Pen for scoring
    - Level floor surface for standing assessment
    - Well-lit examination room

4.2.3 Patient Preparation

1. **Participant positioning:**
    - Remove shoes and socks
    - Stand barefoot on level floor
    - Feet approximately hip-width apart (comfortable natural stance)
    - Weight distributed evenly between both feet
    - Arms relaxed at sides
    - Look straight ahead (not down at feet)
2. **Stance standardization:**
    - Instructed to adopt "relaxed standing" position
    - No correction or manipulation of foot position by evaluator
    - Allow 30 seconds for participant to settle into natural stance

    - Position held stable for approximately 2 minutes during assessment

3. **Environment:**
    - Quiet room free from distractions
    - Good lighting (natural or artificial) to observe foot contours
    - Room temperature comfortable (to avoid muscle tension from cold)

4.2.4 Assessment Procedure - Six Criteria

CRITERION 1: Talar Head Palpation

- **Evaluator position:** Behind participant
- **Technique:**
    - Palpate talar head on both medial and lateral aspects of foot
    - Thumb on medial side, index finger on lateral side
    - Gentle pressure to identify bony prominence
- **Scoring:**
    - **-2:** Talar head palpable on lateral side, not palpable on medial side (inverted/supinated)
    - **-1:** Talar head slightly more palpable on lateral side than medial
    - **0:** Talar head equally palpable on both sides
    - **+1:** Talar head slightly more palpable on medial side than lateral
    - **+2:** Talar head palpable on medial side, not palpable on lateral side (everted/pronated)

CRITERION 2: Curves Above and Below Lateral Malleolus

- **Evaluator position:** Lateral aspect of foot
- **Observation:** Compare curvature of soft tissue immediately above vs. below lateral malleolus
- **Scoring:**
    - **-2:** Curve below malleolus either straight or convex (supinated)
    - **-1:** Curve below malleolus concave but flatter than above

  - **0:** Both curves approximately equal in depth
  - **+1:** Curve below malleolus more concave than above
  - **+2:** Curve below malleolus markedly more concave than above (pronated)

CRITERION 3: Calcaneal Frontal Plane Position

- **Evaluator position:** Directly behind participant
- **Observation:** Assess calcaneal bisection relative to vertical axis
- **Reference:** Imaginary vertical line from ground upward
- **Scoring:**
  - **-2:** Calcaneus inverted >5° from vertical (supinated)
  - **-1:** Calcaneus inverted between vertical and 5°
  - **0:** Vertical (perpendicular to ground, 0°)
  - **+1:** Calcaneus everted between vertical and 5°
  - **+2:** Calcaneus everted >5° from vertical (pronated)

CRITERION 4: Bulge in Region of Talonavicular Joint

- **Evaluator position:** Behind and slightly medial to participant
- **Observation:** Assess for bulging in area of talonavicular joint (medial midfoot)
- **Palpation:** Gentle palpation to confirm visual observation
- **Scoring:**
  - **-2:** Area of TNJ curved evenly with no flattening; concave contour (supinated)
  - **-1:** Area of TNJ essentially flat
  - **0:** Area of TNJ bulging slightly
  - **+1:** Area of TNJ obviously bulging
  - **+2:** Area of TNJ very prominent bulge; convex contour (pronated)

CRITERION 5: Congruence of Medial Longitudinal Arch (MLA)

- **Evaluator position:** Medial aspect of foot
- **Observation:** Assess height and shape of medial longitudinal arch

- **Reference points:** Navicular tuberosity to 1st metatarsal head
- **Scoring:**
  - **-2:** Arch high and acutely angled toward posterior; peak near navicular (supinated)
  - **-1:** Arch moderately high and slightly acute posteriorly
  - **0:** Arch height normal with smooth, even curve
  - **+1:** Arch lowered with some flattening in central portion; curve less pronounced
  - **+2:** Arch very low with severe flattening; ground contact possible in arch area (pronated)

CRITERION 6: Abduction/Adduction of Forefoot on Rearfoot

- **Evaluator position:** Directly behind participant
- **Observation:** Assess alignment of forefoot relative to rearfoot; count visible toes
- **Key indicator:** Number of lateral toes visible from behind
- **Scoring:**
  - **-2:** No toes visible from behind; medial toes may be visible (forefoot adducted/supinated)
  - **-1:** Only medial aspect of 5th toe visible
  - **0:** Medial and lateral aspects of 5th toe equally visible; maybe small portion of 4th toe
  - **+1:** 4th and 5th toes clearly visible from behind
  - **+2:** At least 3rd, 4th, and 5th toes clearly visible (forefoot abducted/pronated)

4.2.5 Scoring and Interpretation

Score Calculation:

- Sum all six individual criterion scores
- Total score range: -12 (highly supinated) to +12 (highly pronated)

Classification:

- **Highly supinated:** -12 to -5
- **Supinated:** -4 to -1
- **Normal:** 0 to +5
- **Pronated:** +6 to +9
- **Highly pronated:** +10 to +12

Time per Participant:

- Approximately 4-5 minutes per foot
- Bilateral assessment: 8-10 minutes total

Key Points for Reliability:

- Same relaxed standing position maintained for all criteria
- No adjustment or correction of participant's natural stance
- Each criterion scored independently (avoid halo effect)
- Systematic approach: always assess criteria in same order (1→6)
- Evaluator always observes from same standardized positions

4.3 Goniometric Assessment Protocol (Shank-Forefoot Alignment)

4.3.1 Background

The shank-forefoot alignment measurement protocol follows the standardized method described by Mendonça et al. (2013), which has demonstrated:

- Excellent intra-rater reliability: ICC 0.90-0.93
- Excellent inter-rater reliability: ICC 0.90-0.91
- Good correlation with photogrammetric methods: r = 0.71 (Diniz et al., 2020)

Key Advantages:

- Does NOT require subtalar neutral positioning (which has poor reliability; Picciano et al., 1993)
- Uses palpable metallic nail as landmark (reduces soft tissue palpation variability)
- Standardized ankle position at 90° dorsiflexion

4.3.2 Equipment

- **Goniometer:** Two-arm 50 cm goniometer (Miskall)
    - Arms: 50 cm length each
    - Scale: 1-degree increments (0-180° protractor)
    - Material: Rigid plastic arms with metal fastener at fulcrum
    - Calibration: Verified against known angles (0°, 45°, 90°, 180°) before study
- **Metallic Nail:**
    - Length: 10 cm
    - Diameter: 2 mm
    - Material: Stainless steel (autoclavable)
    - Purpose: Radiographic marker AND palpable reference for goniometry
    - Sterilization: Autoclaved between participants
- **Additional Equipment:**
    - Examination table (adjustable height)
    - Small pillow for ankle support
    - Alcohol wipes for goniometer cleaning between participants
    - Data recording sheet

4.3.3 Patient Preparation and Positioning

Step 1: Initial Setup

- Participant removes shoes and socks
- Lies prone on examination table
- Knees fully extended (0° flexion)
- Arms comfortable at sides or supporting head

Step 2: Ankle Positioning

- Small pillow placed under anterior ankle (distal tibia/fibula)
- Evaluator passively positions ankle at 90° dorsiflexion

- 90° angle verified by visual inspection (tibia perpendicular to sole of foot)
- Foot hangs freely in space beyond edge of table (no contact with surface)

Step 3: Position Verification

- Ensure knees remain fully extended
- Verify ankle maintained at 90° dorsiflexion
- Check that foot is relaxed (no active muscle contraction)
- Subtalar joint is NOT positioned in neutral (per Mendonça protocol)

Rationale for NOT Using Subtalar Neutral:

- Subtalar neutral positioning shows poor-to-moderate inter-rater reliability (ICC 0.30-0.60; Van Gheluwe et al., 2002)
- Subjective determination based on "feel" introduces measurement variability
- Mendonça protocol avoids this source of error by using fixed ankle dorsiflexion only

4.3.4 Anatomical Landmark Identification

Step 1: Palpation and Marking (Skin Marking Pen)

Tibial Bisector Reference Points:

1. **Proximal reference:** Midpoint of popliteal fossa (knee level)
2. **Middle reference:** Midpoint of calf at maximum girth
3. **Distal reference:** Midpoint between medial and lateral malleoli
4. **Verification:** Three points should form straight line when viewed from behind

Calcaneal Reference Points (for orientation, not direct measurement):

1. Most prominent point of medial calcaneus
2. Most prominent point of lateral calcaneus
3. Posterior prominence of calcaneus

Metatarsal Head Landmarks:

1. Head of 1st metatarsal (most prominent medial point on plantar surface)
2. Head of 5th metatarsal (most prominent lateral point on plantar surface)

3. Palpate to confirm correct location (heads, not shafts or bases)

4.3.5 Metallic Nail Placement

Step 1: Positioning

- Metallic nail placed horizontally under plantar surface of forefoot
- Positioned to simultaneously contact BOTH 1st and 5th metatarsal heads
- Nail perpendicular to long axis of foot

Step 2: Verification

- Palpate to confirm nail in contact with both metatarsal heads
- Visual inspection from medial and lateral aspects
- Check that nail does not rest on metatarsal shafts (only heads)
- Verify horizontal position (not tilted anteriorly or posteriorly)

Step 3: Nail Stabilization

- Evaluator's non-dominant hand gently maintains nail position
- Minimal pressure applied (just enough to prevent nail displacement)
- Participant instructed to keep foot completely relaxed

4.3.6 Goniometer Placement and Angle Measurement

Step 1: Goniometer Axis Placement

- Axis (fulcrum) of goniometer placed at midpoint of ankle
- Precise location: Midway between medial and lateral malleoli
- Axis should align with imaginary transverse line through ankle joint

Step 2: Stationary Arm Alignment

- Stationary arm aligned with tibial bisector
- Arm points upward along posterior calf
- Alignment verified by checking that arm passes through all three previously marked reference points (ankle, mid-calf, knee)

Step 3: Mobile Arm Alignment

- Mobile arm aligned with metallic nail under metatarsal heads
- Arm points downward/forward along plantar surface
- Alignment verified by visual inspection from multiple angles

Step 4: Frontal Plane Verification

- Ensure entire goniometer remains in frontal plane (not tilted medially or laterally)
- Evaluator's line of sight perpendicular to goniometer scale
- Check from behind and from lateral aspect

Step 5: Angle Reading

- Read angle to nearest degree on goniometer scale
- Record immediately on data sheet
- Sign convention:
    - **Positive values:** Forefoot inverted relative to leg (varus)
    - **Negative values:** Forefoot everted relative to leg (valgus)

4.3.7 Three-Measurement Protocol

Rationale: Multiple measurements with complete repositioning reduce random measurement error and improve reliability (Stratford and Goldsmith, 1997).

Procedure:

1. **First Measurement:**
    - Complete measurement as described above
    - Record angle
2. **Complete Repositioning:**
    - Remove goniometer completely
    - Remove metallic nail
    - Participant relaxes foot completely (10-15 seconds)
    - Re-palpate landmarks
    - Replace metallic nail

- Replace goniometer
- Verify all positioning criteria

3. **Second Measurement:**
    - Repeat complete measurement procedure
    - Record angle
4. **Complete Repositioning:**
    - Repeat repositioning sequence
5. **Third Measurement:**
    - Repeat complete measurement procedure
    - Record angle

Data Processing:

- Calculate mean of three measurements
- Mean used for all analyses (per standard recommendations; Stratford and Goldsmith, 1997)
- Individual measurements retained for calculation of measurement variability

Time per Participant:

- Approximately 15-20 minutes for bilateral assessment (both feet)
- Per foot: ~7-10 minutes for three measurements

4.4 Radiographic Assessment Protocol

4.4.1 Radiation Safety

ALARA Principle: All radiographic procedures followed "As Low As Reasonably Achievable" (ALARA) principles for radiation protection:

- Minimum number of exposures: 1 per foot
- Total exposures per participant: 2
- Optimized exposure settings: 60kV, 2.5mA, 0.1s (lowest settings providing diagnostic quality images)

- Collimation: Beam restricted to foot/ankle region only
- Gonadal shielding: Applied when anatomically feasible without obscuring foot
- Pregnancy exclusion: All females of childbearing age screened

Radiation Dose Estimates:

- Effective dose per exposure: ~0.001 mSv (milliSieverts)
- Total effective dose per participant: ~0.004 mSv (4 exposures)
- Comparison:
    - Background radiation (1 day): ~0.008 mSv
    - Chest X-ray: ~0.02 mSv
    - Current study: Equivalent to ~0.5 days of background radiation

Informed Consent: Participants explicitly informed about:

- Radiation exposure involved
- Estimated dose and comparison to common exposures
- Risks (minimal at this dose level)
- Right to decline radiographic portion while continuing with goniometric assessment

4.4.2 Equipment and Settings

X-ray System:

- **Model:** Siemens POLYMOBIL III/Plus portable radiographic system
- **Manufacturer:** Siemens Healthcare GmbH, Erlangen, Germany
- **Type:** Mobile C-arm fluoroscopy system adapted for static radiography
- **Detector:** Digital flat-panel detector

Imaging Software:

- **Software:** VistaScan Plus Ceph
- **Manufacturer:** Dürr Dental, Bietigheim-Bissingen, Germany
- **Version:** 2.11
- **Capabilities:**

- Digital angle measurement tools (protractor function)
- Line drawing for landmark identification
- Precise angle calculation (0.1° precision)
- Image calibration and scaling
- Data export for analysis

Standardized Exposure Settings:

- **Tube voltage (kVp):** 60 kV
  - Rationale: Optimal for foot/ankle imaging (adequate penetration, good contrast)
- **Tube current (mA):** 2.5 mA
  - Rationale: Sufficient for diagnostic image quality with digital detector
- **Exposure time:** 0.1 seconds
  - Rationale: Minimizes motion artifact while maintaining image quality
- **Source-to-image distance (SID):** 100 cm (fixed for all acquisitions)
  - Rationale: Standard distance for extremity imaging; minimizes magnification
- **Collimation:** Restricted to foot/ankle region
- **Focal spot size:** 0.6 mm

Calibration:

- X-ray equipment: Annual calibration by certified technician
- Software calibration: Monthly verification using phantom with known angles
- Daily image quality check: Test pattern exposure before clinical use

4.4.3 Patient Positioning for Radiography

Position: Prone (same as goniometry)

Advantages of Prone Position:

- Consistent with clinical goniometric measurement position
- Natural foot position without weight-bearing distortion
- Ankle can be maintained at 90° dorsiflexion easily

- Reduces patient anxiety (cannot see X-ray equipment)

Setup:

1. Participant remains prone on radiolucent table
2. Ankle at 90° dorsiflexion (verified with positioning aid/block)
3. Foot elevated on radiolucent foam block (if needed for optimal X-ray beam alignment)
4. Foot relaxed (no active muscle contraction)
5. Lead shielding positioned over pelvic region

4.4.4 Radiographic Method 1: Marker-Based Method

Concept: Uses two external radiopaque markers placed at specific anatomical landmarks on skin surface. Based on Heidelberg foot measurement protocol (Schaefer et al., 2016).

Markers:

- **Type:** Small metallic spheres (lead-based)
- **Size:** 5 mm diameter
- **Attachment:** Medical-grade double-sided adhesive tape
- **Radiopacity:** High density; clearly visible on radiographs as circular shadows

Marker Placement:

Marker 1 (Medial):

- Location: Midpoint of 1st metatarsal head
- Palpation technique:
    - Identify most prominent plantar point of 1st metatarsal head.
    - Mark with pen.
    - Apply marker over mark using adhesive tape
- Verification: Gentle palpation through marker confirms correct placement

Marker 2 (Lateral):

- Location: Midpoint of 5th metatarsal head
- Palpation technique:

    - Identify most prominent plantar point of 5th metatarsal head
    - Mark with pen
    - Apply marker over mark using adhesive tape
- Verification: Gentle palpation through marker confirms correct placement

Tibial Reference Marker:

- Location: Posterior ankle at level of ankle joint center
- Placed on skin over midpoint between malleoli
- Purpose: Marks proximal end of tibial bisector line

X-ray Beam Positioning:

- **Direction:** Anterior-to-posterior (AP) projection
- **Central ray:** Directed perpendicular to plantar surface of foot
- **Aimed at:** Level of ankle joint
- **Source-to-image distance:** 100 cm (measured with laser rangefinder)

Image Acquisition:

- Single exposure per foot with markers in place
- Participant instructed to remain completely still
- Breath holding not required (extremity imaging)
- Exposure triggered by radiographer

Image Quality Verification:

- Immediate review of digital image on monitor
- Verify:
    - Both markers clearly visible
    - Tibial reference visible
    - Adequate contrast and resolution
    - No motion artifact
- Repeat exposure if quality inadequate (occurred in 0 cases)

4.4.5 Radiographic Method 2: Nail-Based Method

Concept: Uses the same metallic nail employed in clinical goniometry as radiopaque reference. Nail remains in place under metatarsal heads from goniometric assessment. Originally described by Mendonça et al. (2013).

Nail Specifications:

- Same nail used for goniometry (already in position)
- Length: 10 cm
- Diameter: 2 mm
- Material: Stainless steel (radiopaque)
- Position: Horizontal under plantar surface, contacting both 1st and 5th metatarsal heads

X-ray Beam Positioning:

- Identical to marker-based method
- **Direction:** Anterior-to-posterior projection
- **Central ray:** Perpendicular to plantar surface
- **SID:** 100 cm

Image Acquisition:

- Single exposure per foot with nail in place
- Same exposure settings as marker method (60kV, 2.5mA, 0.1s)
- Participant remains still during exposure

Advantages of Nail Method:

- Nail already positioned from goniometry (no additional time)
- Same reference used for clinical and radiographic measurement (direct comparison)
- Continuous metallic line easier to identify on radiograph than discrete points

Limitations of Nail Method:

- Nail may cast shadow on underlying bony structures
- Nail position dependent on soft tissue compression
- Less anatomically precise than markers directly on bony landmarks

4.4.6 Digital Angle Measurement

All radiographic angles measured by single experienced radiologist (V.O.) using VistaScan Plus Ceph software.

Measurement Procedure for Marker-Based Method:

Step 1: Image Display

- Open digital radiograph in measurement software
- Adjust brightness and contrast for optimal landmark visualization
- Zoom to appropriate level (typically 100-150% magnification)

Step 2: Tibial Bisector Identification

- Identify proximal tibia in image
- Identify ankle joint center (midpoint between malleoli)
- Draw digital line (software line tool) from proximal tibia through ankle center
- Line represents tibial bisector (mechanical axis of leg)
- Verify line passes through center of distal 1/3 of tibia

Step 3: Forefoot Reference Line

- Identify the two radiopaque markers (1st and 5th metatarsal heads)
- Draw digital line connecting centers of both markers
- This line represents forefoot reference (plane of metatarsal heads)

Step 4: Angle Calculation

- Use software protractor tool
- Place protractor vertex at intersection of tibial bisector and forefoot reference line
- Software automatically calculates angle between the two lines
- Angle displayed with 0.1° precision

Step 5: Sign Convention

- Software displays acute angle (0-90°)
- Evaluator assigns sign based on relationship:

- **Positive (+):** Forefoot inverted relative to leg (varus)
- **Negative (-):** Forefoot everted relative to leg (valgus)

- Record angle with sign on data sheet

Measurement Procedure for Nail-Based Method:

Identical to marker method except:

- Instead of connecting two discrete markers, draw line along the radiopaque nail
- Nail appears as continuous linear radiopaque shadow
- Draw line through center of nail shadow
- All other steps identical

Intra-Rater Reliability Procedure (Radiographic):

- Radiologist (V.O.) repeated angle measurements on follow-up radiographs (n=23)
- Minimum 30-day interval between measurements
- Radiologist blinded to previous measurements (stored separately)
- No inter-rater radiographic reliability assessed (see justification in main text)

Time per Participant:

- Image acquisition: ~10 minutes (4 exposures total, 2 per foot)
- Image analysis (performed later): ~5 minutes per participant (8 angles measured)

---

5. EQUIPMENT SPECIFICATIONS

5.1 Goniometer

Model: Two-arm 50 cm goniometer **Manufacturer:** Miskall (Spain) **Specifications:**

- **Arm length:** 50 cm each arm (100 cm total span when fully extended)
- **Scale type:** 360° protractor with 1-degree increments
- **Scale accuracy:** ±1° (verified against precision angle gauge)
- **Material:** Rigid transparent plastic arms with stainless steel fastener at fulcrum
- **Arm width:** 2.5 cm (provides stable placement on limb segments)

- **Transparency:** Clear plastic allows visualization of underlying anatomy
- **Durability:** Impact-resistant plastic suitable for clinical use

Calibration:

- **Pre-study calibration:** Verified against known angles (0°, 45°, 90°, 135°, 180°) using precision angle gauge
- **Calibration results:** All readings within ±1° of true angles
- **During study:** Visual inspection before each session for any damage or loosening of fulcrum
- **Cleaning:** Alcohol wipe disinfection between participants

Maintenance:

- Fulcrum tightness checked weekly
- Any goniometer showing >2° deviation from known angles removed from use
- No goniometers required replacement during study period

5.2 Metallic Nail

Specifications:

- **Length:** 10 cm
- **Diameter:** 2 mm
- **Material:** Stainless steel (medical grade 316L)
- **Surface:** Smooth polished finish
- **Sterility:** Autoclavable at 134°C, 15 minutes
- **Radiopacity:** Sufficient for clear visualization on radiographs at study exposure settings

Purpose:

- **Dual function:**
    - Tactile reference landmark for clinical goniometry
    - Radiopaque marker for radiographic measurement

- **Advantages:**
    - Same reference used for both measurement methods
    - Enables direct comparison between clinical and radiographic angles

Procurement:

- Custom-manufactured stainless steel rods
- Cut to 10 cm length
- Ends smoothed (no sharp edges)
- Quantity: 5 nails (for rotation between sterilization cycles)

Sterilization Protocol:

- Autoclaved after each use
- Stored in sterile packaging
- Single-use per participant (no re-use without sterilization)

5.3 Radiographic Equipment

X-ray Unit:

- **Model:** Siemens POLYMOBIL III/Plus
- **Type:** Mobile radiographic system
- **Manufacturer:** Siemens Healthcare GmbH, Erlangen, Germany
- **Year:** 2018
- **Specifications:**
    - **Output voltage range:** 40-125 kV
    - **Output current range:** 0.1-10 mA
    - **Focal spot size:** 0.6 mm (fine focus)
    - **Tube type:** Fixed anode
    - **Collimation:** Adjustable light beam diaphragm
    - **Mobility:** Wheeled base with position lock

Digital Imaging System:

- **Detector type:** Flat-panel digital detector
- **Detector size:** 30 cm × 40 cm
- **Pixel matrix:** 2880 × 2304 pixels
- **Pixel size:** 0.104 mm
- **Gray scale:** 16-bit (65,536 shades)
- **Detective quantum efficiency (DQE):** 70% at 1 lp/mm

Software:

- **Name:** VistaScan Plus Ceph
- **Manufacturer:** Dürr Dental, Bietigheim-Bissingen, Germany
- **Version:** 2.11
- **Operating system:** Windows 10 Professional
- **Measurement tools:**
    - Digital protractor with 0.1° precision
    - Line drawing tool with adjustable thickness
    - Angle calculation (automatic between intersecting lines)
    - Distance measurement with calibration
    - Zoom function (25-400%)
    - Brightness and contrast adjustment
    - Image export (DICOM, JPEG, PNG formats)

Calibration:

- **X-ray equipment calibration:**
    - Annual calibration by certified Siemens technician
    - Includes: kV accuracy, mA accuracy, exposure timer, focal spot size, collimation accuracy
    - Last calibration: December 2023

- All parameters within manufacturer specifications

- **Software calibration:**
  - Monthly: Phantom with known angles (0°, 15°, 30°, 45°, 90°)
  - Software angle measurements verified against phantom
  - Acceptance criteria: Within ±0.2° of true angle
  - All monthly checks passed during study period
- **Daily quality control:**
  - Test pattern exposure each morning
  - Visual inspection: Image quality, contrast, spatial resolution
  - All daily checks passed during study period

Radiation Monitoring:

- **Dosimetry:** Personal dosimeters worn by radiographer
- **Area monitoring:** Portable survey meter used to verify scatter radiation
- **Participant exposure documentation:** Recorded in hospital radiation log

---

5.4 Radiopaque Markers

Specifications:

- **Type:** Spherical lead markers
- **Diameter:** 5 mm
- **Material composition:**
  - Core: Lead (for radiopacity)
  - Coating: Thin plastic shell (biocompatible, smooth surface)
- **Weight:** ~0.5 grams each
- **Radiopacity:** High density; visible on radiographs as well-defined circular shadows
- **Reusability:** Single-use per participant

Adhesive:

- **Type:** Medical-grade double-sided adhesive tape
- **Manufacturer:** 3M Healthcare
- **Properties:**
    - Hypoallergenic
    - Strong initial tack
    - Clean removal without skin irritation
    - Residue-free

Quantity:

- 2 markers per set
- Fresh markers used for each participant

Procurement:

- Custom-made for study purposes
- Sourced from medical device supplier
- CE marked for medical use

5.5 Foot Posture Index (FPI-6) Materials

Equipment Required:

- **None** (purely observational clinical tool)

Materials:

- **FPI-6 data recording sheet:**
    - Spanish language version
    - Contains:
        - Patient identifier
        - Date of assessment
        - Scoring boxes for each of 6 criteria (left and right foot)
        - Total score calculation boxes

        - Classification result (supinated/normal/pronated)
        - Evaluator signature
- **Writing instrument:** Pen for scoring
- **Reference materials:**
    - FPI-6 instruction manual (Redmond et al., 2006)
    - Visual reference guide with scoring examples

Environmental Requirements:

- Level floor surface (verified with spirit level)
- Adequate lighting: Minimum 500 lux at floor level
- Room temperature: 20-24°C (comfortable for barefoot standing)
- Quiet environment free from distractions

5.6 Additional Materials

Data Recording:

- Standardized data collection forms (paper)
- Pencils and pens
- Clipboard
- Electronic database (Microsoft Excel) for data entry

Patient Comfort:

- Examination table with adjustable height
- Pillow for ankle support during prone positioning
- Clean linens changed between participants
- Patient gown (if requested)

Hygiene:

- Alcohol wipes (70% isopropyl) for goniometer disinfection
- Hand sanitizer (for evaluators)

- Disposable paper towels

Safety:

- Lead apron (for radiographer)
- Lead shields (for gonadal protection of participants)
- Emergency contact information displayed
- First aid kit available

---

6. GRRAS CHECKLIST COMPLIANCE

Complete GRRAS Checklist with Location in Manuscript

GRRAS Item	Requirement	Location in Manuscript	Compliance Status
**1. Title/Abstract**	Identify reliability/agreement study	Title, Abstract	✅ Complete
**2. Device description**	Name and describe measurement tools	Methods - Equipment; Supplementary Section 5	✅ Complete
**3. Subject population**	Specify population of interest	Methods - Participants; Introduction	✅ Complete
**4. Rater population**	Specify rater characteristics	Methods - Study Design; Supplementary Section 1	✅ Complete
**5. Rationale**	Describe what is known and study justification	Introduction	✅ Complete
**6. Sample size**	Explain calculation, state planned numbers	Methods - Sample Size; Supplementary Section 2	✅ Complete
**7. Sampling method**	Describe sampling approach	Methods - Participants; Supplementary Section 3	✅ Complete

GRRAS Item	Requirement	Location in Manuscript	Compliance Status
**8. Rating process**	Describe procedures, intervals, blinding	Methods - Study Design, Data Acquisition; Supplementary Section 4	✅ Complete
**9. Independence**	State whether ratings conducted independently	Methods - Study Design	✅ Complete
**10. Statistical analysis**	Describe statistical methods	Methods - Statistical Analysis	✅ Complete
**11. Actual numbers**	Report actual n of raters, subjects, observations	Methods - Participants, Results	✅ Complete
**12. Sample characteristics**	Describe raters and subjects	Methods - Participants, Study Design; Supplementary Section 1	✅ Complete
**13. Report estimates**	Report ICC/agreement with confidence intervals	Results	✅ Complete
**14. Practical relevance**	Discuss clinical/practical implications	Discussion	✅ Complete
**15. Detailed results**	Provide comprehensive data	Results, Supplementary Material	✅ Complete

---

Detailed GRRAS Compliance Statement

Item 1 (Title and Abstract):

- Title explicitly states "Reliability and Agreement"
- Abstract clearly identifies study as reliability assessment
- Keywords include "Reproducibility of results"

Item 2 (Measurement Device Description):

- Main text: Brief description of all measurement tools

- Supplementary Material Section 5: Complete specifications including:
    - Goniometer: Make, model, specifications, calibration
    - Radiographic system: Complete technical details
    - Markers: Composition, size, attachment method
    - FPI-6: Validated tool with reference

Item 3 (Subject Population):

- Main text: Recruitment source, inclusion criteria
- Supplementary Section 3: Detailed recruitment procedures, screening results
- Population: Adults (≥18 years) able to achieve 90° ankle dorsiflexion

Item 4 (Rater Population):

- Main text: Rater experience levels (expert vs. novice), training duration
- Supplementary Section 1: Complete rater profiles including qualifications, experience, training
- Clear differentiation: Expert (15 years with protocol) vs. Novice (extensive clinical experience but new to protocol)

Item 5 (Rationale and Literature Review):

- Introduction: Comprehensive review of existing methods
- Prevalence variability discussed (8.6%-83.67%)
- Gaps in knowledge identified
- Citation of previous systematic review (Carrelero Camp et al., 2026)
- Justification for radiographic validation provided

Item 6 (Sample Size Calculation):

- Main text: Parameters stated (ρ_0=0.65, ρ_1=0.85, α=0.005, power=80%)
- Supplementary Section 2: Complete calculations using Walter et al. (1998) method
- Justification for parameters provided
- Actual vs. planned sample size addressed

Item 7 (Sampling Method):

- Main text: Consecutive sampling explicitly stated
- Supplementary Section 3: Detailed screening flow
- Rationale for consecutive sampling provided
- Screening results and enrollment rates reported

Item 8 (Measurement/Rating Process):

- Main text: Session structure, time intervals, blinding procedures
- Supplementary Section 4: Step-by-step protocols for all measurements
- Detailed description of:
    - Patient positioning
    - Landmark identification
    - Equipment placement
    - Angle reading procedures
    - Standardization methods

Item 9 (Independence of Measurements):

- Main text: Explicitly states "independently by two evaluators"
- Blinding confirmed: "Evaluators were blinded to each other's measurement"
- 30-minute interval between evaluators ensures temporal independence

Item 10 (Statistical Analysis):

- Main text: Detailed statistical methods section including:
    - ICC models specified: ICC(2,1) and ICC(3,1)
    - Formulas for SEM and MDC provided
    - Interpretation criteria stated
    - Software identified (SPSS 27, RStudio)
    - Significance threshold justified ($p<0.005$)

Item 11 (Actual Numbers):

- Main text:

  - Participants: 35 (70 limbs)
  - Intra-rater subsample: 23 (46 limbs)
  - Raters: 2 goniometric + 1 radiographic
  - Observations: 3 measurements per evaluator per foot
- Supplementary Section 3: Complete enrollment flow

Item 12 (Sample Characteristics):

- Main text: Age (32.7±10.8 years), sex (54% female), BMI (23.3±3.3)
- Classification: 97.1% FV, 2.9% valgus
- Rater characteristics: Experience levels, training completion
- Supplementary Sections 1 & 3: Complete demographic and clinical profiles

Item 13 (Report Estimates with Uncertainty):

- Results section reports:
  - ICC values with 95% confidence intervals
  - SEM and MDC95 values
  - Bland-Altman limits of agreement
  - Correlation coefficients with confidence intervals

Item 14 (Practical Relevance):

- Discussion addresses:
  - Clinical utility of each method
  - Interpretation of MDC values for clinical practice
  - Recommendations for method selection based on purpose
  - Cost-effectiveness considerations

Item 15 (Detailed Results/Auxiliary Material):

- Supplementary Material provides:
  - Complete methodology details
  - Training protocol specifics

    - Equipment specifications
    - Sample size calculations
    - Detailed measurement protocols
- Raw data available upon reasonable request

---

7. REFERENCES

1. Redmond, A.C., Crosbie, J., Ouvrier, R.A., 2006. Development and validation of a novel rating system for scoring standing foot posture: the Foot Posture Index. Clinical Biomechanics 21(1), 89-98.
2. Mendonça, L.D.M., Bittencourt, N.F.N., Amaral, G.M., Diniz, L.S., Souza, T.R., Fonseca, S.T., 2013. A quick and reliable procedure for assessing foot alignment in athletes. Journal of the American Podiatric Medical Association 103(5), 405-410.
3. Van Gheluwe, B., Kirby, K.A., Roosen, P., Phillips, R.D., 2002. Reliability and accuracy of biomechanical measurements of the lower extremities. Journal of the American Podiatric Medical Association 92(6), 317-326.
4. Kottner, J., Audigé, L., Brorson, S., Donner, A., Gajewski, B.J., Hróbjartsson, A., Roberts, C., Shoukri, M., Streiner, D.L., 2011. Guidelines for Reporting Reliability and Agreement Studies (GRRAS) were proposed. Journal of Clinical Epidemiology 64(1), 96-106.
5. Walter, S.D., Eliasziw, M., Donner, A., 1998. Sample size and optimal designs for reliability studies. Statistics in Medicine 17(1), 101-110.
6. Shrout, P.E., Fleiss, J.L., 1979. Intraclass correlations: Uses in assessing rater reliability. Psychological Bulletin 86(2), 420-428.
7. Streiner, D.L., Norman, G.R., 2008. Health Measurement Scales: A Practical Guide to Their Development and Use, 4th edition. Oxford University Press, Oxford.
8. Kottner, J., Dassen, T., 2008. Interpreting interrater reliability coefficients of the Braden scale: A discussion paper. International Journal of Nursing Studies 45(8), 1239-1246.
9. Diniz, K.M.A., Mascarenhas, R.O., Freire, R., Bittencourt, N.F.N., Mendonça, L.D.M., 2020. Correlation between goniometric and photogrammetric assessment of shank-forefoot alignment in athletes. Foot 45, 101687.

10. Picciano, A.M., Rowlands, M.S., Worrell, T., 1993. Reliability of open and closed kinetic chain subtalar joint neutral positions and navicular drop test. Journal of Orthopaedic & Sports Physical Therapy 18(4), 553-558.

11. Elveru, R.A., Rothstein, J.M., Lamb, R.L., 1988. Goniometric reliability in a clinical setting. Subtalar and ankle joint measurements. Physical Therapy 68(5), 672-677.

12. Stratford, P.W., Goldsmith, C.H., 1997. Use of the standard error as a reliability index of interest: An applied example using elbow flexor strength data. Physical Therapy 77(7), 745-750.

13. Shoukri, M.M., 2004. Measures of Interobserver Agreement. Chapman & Hall/CRC, Boca Raton, FL.

14. Menz, H.B., Dufour, A.B., Katz, P., Hannan, M.T., 2013. Foot pain and pronated foot type are associated with self-reported mobility limitations in older adults: The Framingham Foot Study. Gerontology 62(3), 289-295.

15. Evans, A.M., Copper, A.W., Scharfbillig, R.W., Scutter, S.D., Williams, M.T., 2003. Reliability of the Foot Posture Index and traditional measures of foot position. Journal of the American Podiatric Medical Association 93(3), 203-213.

16. Redmond, A.C., Crane, Y.Z., Menz, H.B., 2008. Normative values for the Foot Posture Index. Journal of Foot and Ankle Research 1(1), 6.

17. Schaefer, I., Biehlmann, C., Valderrabano, V., Easley, M.E., Wiewiorski, M., 2016. Radiographic assessment of foot alignment in adult acquired flatfoot deformity. Foot & Ankle International 37(3), 298-305.

18. Colquhoun, D., 2014. An investigation of the false discovery rate and the misinterpretation of p-values. Royal Society Open Science 1(3), 140216.
