# Supplementary material for: Reliability and Agreement of a Dual-Method Radiographic Standard vs. Clinical Goniometry for Shank–Forefoot Alignment: A GRRAS-Compliant Study
Source: Diagnostics (Basel). 2026 Feb 27;16(5):703. doi: 10.3390/diagnostics16050703 (PMC12984665; doi:10.3390/diagnostics16050703)
Supplement: Supplementary file 1 [file diagnostics-16-00703-s001.zip › File S4. Supplementary material within-participant correlation.pdf]

Supplementary material within-participant correlation

Sensitivity analysis comparing reliability metrics between full bilateral dataset (n=70 limbs from 35 participants) and randomly selected single limb per participant (n=35).

For the n=35 analysis, one limb per participant was randomly selected using Excel's random number generation function. ICC = Intraclass correlation coefficient; CI = Confidence interval; SEM = Standard error of measurement (degrees); MDC95 = Minimal detectable change at 95% confidence (degrees); CV = Coefficient of variation (%). All ICC values remained excellent (>0.90) and all findings remained statistically significant (p<0.005) regardless of dataset size.

Measurement	Dataset	ICC	95% CI Lower	95% CI Upper	SEM	MDC95	CV (%)
**Goniometry inter-rater**	n=70	0.987	0.977	0.993	0.017	0.364	0.974
	n=35	0.989	0.976	0.995	0.005	0.190	0.295
**Marker-based radiography intra-rater**	n=70	0.906	0.852	0.944	0.166	1.129	3.672
	n=35	0.952	0.905	0.976	0.089	0.827	—
**Inter-method agreement (Gonio vs Marker)**	n=70	0.898	0.843	0.938	0.151	1.079	—
	n=35	0.933	0.878	0.968	0.166	1.130	—
**FPI inter-rater**	n=70	0.951	0.913	0.972	0.062	0.689	5.551
	n=35	0.946	0.879	0.976	0.061	0.687	5.019

The sensitivity analysis conclusively demonstrated that our findings are robust and not artifacts of within-participant correlation. All key reliability coefficients remained in the excellent range with minimal variation from the full dataset (changes in ICC values ranged from -0.002 to +0.046), and all statistical conclusions remained unchanged at the p<0.005 significance level. This confirms the validity of our primary conclusions regarding the reliability and agreement of the measurement methods.

The following tables present these results, maintaining the same structure as the corresponding main manuscript tables (n=70) to facilitate direct comparison. All key findings and statistical inferences remained unchanged in this sensitivity analysis, confirming the validity of our conclusions.

Table S2

	ICC(2,1)	IC95%	SEM	MDC95	CV
Mark 1 – Mark 2	0,664	[0.355; 0.842]	0,707	2,331	3,860
Nail 1 – Nail 2	0,793	[0.571; 0.906]	0,000	0,000	235,988
Mark 1 – Nail 1	0,908	[0.826; 0.953]	0,123	0,973	2,899
Mark 1 – Nail 2	0,732	[0.466; 0.877]	0,283	1,475	3,696
Mark 2 – Nail 1	0,682	[0.383; 0.851]	19,624	12,279	241,010
Mark 2 – Nail 2	0,965	[0.9198; 0.985]	0,006	0,213	0,208
Mark 1,2, Nail 1,2	0,794	[0.658; 0.895]	0,000	0,000	5,055

Table S3

	ICC(2,1)	IC95%	SEM	MDC95
Mark 1 – Mark 2	0,798	[0.524; 0.914]	0,257	1,405
Nail 1 – Nail 2	0,884	[0.727; 0.951]	11,590	9,437
Mark 1 – Nail 1	0,952	[0.905; 0.976]	0,089	0,827
Mark 1 – Nail 2	0,845	[0.635; 0.934]	0,215	1,286
Mark 2 – Nail 1	0,811	[0.554; 0.920]	15,129	10,781
Mark 2 – Nail 2	0,982	[0.958; 0.992]	0,004	0,181
Mark 1,2, Nail 1,2	0,939	[0.885; 0.971]	0,182	1,184

Table S5

FPI Researcher 1 -Researcher	ICC(3,1)	IC95%	SEM	MDC95	CV

Individual measures	0,946	[0.879; 0.976]	0,061	0,687	5,019
Mean measures	0,972	[0.936; 0.988]	0,044	0,583	

Table S6

Shank forefoot Researcher 1 - Researcher 2	ICC(3,1)	IC95%	SEM	MDC95	CV
Individual measures	0,989	[0.976; 0.995]	0,005	0,190	0,295
Mean measures	0,995	[0.988; 0.998]	0,003	0,156	

Table S8

Shank forefoot, Mark 1,2 and Nail 1,2	ICC(3,1)	IC95%	SEM	MDC95	CV
Individual measures	0,737	[0.5898; 0.859]	0,329	1,590	4,398
Mean measures	0,933	[0.878; 0.968]	0,166	1,130	

Table S9

	ICC(3,1)	IC95%	SEM	MDC95	CV
Mark 1 – Shank forefoot	0,572	[0.3; 0.7598]	0,164	1,123	1,734
Mark 2 – Shank forefoot	0,718	[0.441; 0.870]	0,267	1,433	3,384
Nail 1 - Shank forefoot	0,556	[0.278; 0.748]	0,438	1,834	4,632
Nail 2 – Shank forefoot	0,684	[0.386; 0.852]	0,269	1,438	3,224

Table S10

	ICC(3,1)	IC95%	SEM	MDC95
Mark 1 – Shank forefoot	0,728	[0.461; 0.863]	0,131	1,003
Mark 2 – Shank forefoot	0,836	[0.612; 0.930]	0,204	1,251
Nail 1 - Shank forefoot	0,715	[0.435; 0.856]	0,351	1,642
Nail 2 – Shank forefoot	0,812	[0.557; 0.920]	0,207	1,263
